# Use of Modern Regression Analysis in the Dielectric Properties of Foods

**DOI:** 10.3390/foods9101472

**Published:** 2020-10-15

**Authors:** Yu-Kai Weng, Jiunyuan Chen, Ching-Wei Cheng, Chiachung Chen

**Affiliations:** 1 Department of Bio-Industrial Mechanics Engineering, National Chung Hsing University, 250 Kuokung Road, Taichung 40227, Taiwan; ykweng@dragon.nchu.edu.tw; 2 Africa Research Center, National Chung Hsing University, 250 Kuokung Road, Taichung 40227, Taiwan; kepler0953@gmail.com; 3 College of Intelligence, National Taichung University of Science and Technology, Taichung 40427, Taiwan; cwcheng@nutc.edu.tw

**Keywords:** dielectric properties, frequency, moisture content, temperature, regression analysis

## Abstract

The dielectric properties of food materials is used to describe the interaction of foods with electromagnetic energy for food technology and engineering. To quantify the relationship between dielectric properties and influencing factors, regression analysis is used in our study. Many linear or polynomial regression equations are proposed. However, the basic assumption of the regression analysis is that data with a normal distribution and constant variance are not checked. This study uses sixteen datasets from the literature to derive the equations for dielectric properties. The dependent variables are the dielectric constant and the loss factor. The independent variables are the frequency, temperature, and moisture content. The dependent variables and frequency terms are transformed for regression analysis. The effect of other qualitative factors, such as treatment method and the position of subjects on dielectric properties, are determined using categorical testing. Then, the regression equations can be used to determine which influencing factors are important and which are not. The method can be used for other datasets of dielectric properties to classify influencing factors, including quantitative and qualitative variables.

## 1. Introduction

The dielectric properties of foods are the basic information for the interaction of foods with electromagnetic energy. These properties are used to determine the effect of heating and pasteurization treatments. The frequencies that are used are 13.56, 27.12, and 40.68 MHz for radio frequency and 915 and 2450 MHz for microware electric fields. Then, the dielectric properties of foods are used to design successful treatments [1].

The property that is used to describe the dielectric properties of a material is the relative complex property, $\varepsilon^{*}$.

The relative complex property is expressed as in Equation (1):

(1) $$\varepsilon^{\prime \prime }=\varepsilon^{\prime }-j\varepsilon^{\prime \prime }$$

where *ε*′ is the dielectric constant and $\varepsilon^{\prime \prime }$ is the loss factor.

The dielectric constant *ε*′ is the measure of the ability of a material to store energy, which is affected by the electric field. The dielectric loss factor $\varepsilon^{\prime \prime }$ is the measure of the ability of a material to dissipate energy. The thermal energy of the material is converted as in Equation (2):

(2) $$\mathrm{Pv}=E^{2}\sigma =55.63\times 10^{-12}\;f\times E^{2}\times \varepsilon^{\prime \prime }$$

where Pv is the energy that is developed per unit volume in W/m^3^, *f* is the frequency in Hz, and E is the electric field strength inside the load in V/m.

Several techniques are developed to determine the dielectric properties of foods [2,3,4]. Detailed reviews of this topic and the relationship between the dielectric properties have been undertaken [1,5,6,7]. Studies have been published on the dielectric properties of foods such as egg with powder [8], egg whites and whole eggs [9,10], chickpea flour [11], cowpea weevil, black-eyed peas and mung beans [12], butter [13], bread [14], cheese [15], salmon and sturgeon [16], salmon fillets [17], macadamia nuts [18], and pecan kernels [19].

To develop thermal treatments for post-harvest insect control, the dielectric properties of five fruits, two nuts, and four insect larvae at four frequencies and five temperatures were tested and reported [20].

Recently, Routray and Orsat [21] mention the challenges of the application for dielectric properties of foods. The nonuniform heating limits the microwave application of the large-scale processing. The prediction equations of low moisture products such as spices are required to develop the in-package pasteurization technique. The finite element method provides a way to observe the heating patterns, and the prediction equations of dielectric properties are the basic information. The dielectric properties of complex food equations are the concern of food engineers [21]. The development of the prediction equation for the dielectric properties of multi-component foods by their components and its individual prediction equation would be very useful for the food industry.

The factors that affect the dielectric properties of foods include the applied frequency, the temperature, the bulk density, and the concentration and constituents of foods [1,4,5,6,7]. The equations for dielectric properties describe the relationship between the dielectric properties and influencing factors. Calay et al. [22] described three types of prediction equations (Equations (3)–(6)):

1. Variables including temperature, moisture content, and frequency
  (3) $$y_{1}=a_{0}+a_{1}T+a_{2}X+a_{3}f$$
  where $y_{1}$ is the dielectric constant or the dielectric loss factor, T is the temperature in °C, X is the moisture content (wet basis, w.b. or dry basic, d.b.) in %, and f is the applied frequency in MHz.
2. Variables including temperature and moisture content
  (4) $$y_{2}=b_{0}+b_{1}T+b_{2}X+b_{12}T\times X$$
  (5) $$y_{3}=c_{0}+c_{1}T+c_{2}X$$
3. Only one variable with other factors fixed
  (6) $$y_{4}=c_{0}+c_{1}T+c_{2}T^{2}\;\mathrm{at}\;\mathrm{fixed}\;\mathrm{moisture}\;\mathrm{content}\;\mathrm{and}\;\mathrm{frequency}$$

Calay et al. [22] used the coefficient of determination $R^{2}$ as the sole criterion to determine the fitting agreement for these equations.

The published models using data that involve more variables are listed in Table 1. Most of these equations are multiple regression equations. Many linear or polynomial regression equations are established at a fixed frequency for other factors [8,13,14,17,23]. Everard et al. [15] used a partial least square regression to establish empirical equations to predict moisture content. However, the assumption of the partial least square regression in terms of the multi-collinearity was not considered. Guo et al. [10] proposed prediction equations that use the logarithmic form of dielectric properties (dependent variable) and the logarithmic form of influencing factors (independent variable).

Simple linear regression is used widely. These equations proposed by Kannan et al. [24] involved a higher-order polynomial equation with an inverse form of the frequency and an exponential function for other variables. Multiple linear regression models involving the influencing factors, the interaction between these factors, and the density at fixed frequency were used by Zhu et al. [25]. Boldor et al. [26] adopted the power form of dielectric properties to quantify the effect of the moisture content and temperature at fixed frequency. Zhu and Guo [29] proposed a simple linear equation to describe the relationship between dielectric properties and the logarithmic form of the frequency at fixed conditions of the moisture content and temperature for potato starch. Yu et al. [27] proposed a multiple regression model with three factors as independent variables (temperature, moisture content, and frequency) and their interactions between factors.

Most studies use *R*^2^ as the sole criterion. Some studies used the *p*-value to assess the utility of a model [7,19,26,28,29,31]. However, these criteria ($R^{2}$ and *p*-value) are used for the classical regression, not for the modern regression. Using only the $R^{2}$ or *p*-value does not decide the applicability of regression models [31,32,33].

Modern regression expresses the quantitative relationship between dependent variables and influencing factors for a biological system [34,35,36]. In this study, this modern regression technique is used to determine the effect of various factors on the dielectric properties. Modern regression techniques, such as the normal test, the constant variance test, tests on a single regression coefficient, and categorical testing are applied in the study of dielectric properties of foods.

To the best knowledge of the authors, modern regression equations have not been used in a study of dielectric properties. This study determines the usefulness of the modern analysis to determine the influencing factors for the dielectric properties of foods using data from previous studies.

## 2. Materials and Methods

### 2.1. Regression Analysis

A typical multiple linear regression model involves three variables (Equation (7)):

(7) $$y_{i}=b_{0}+b_{0}x_{1}+b_{2}x_{2}+b_{3}x_{3}+b_{11}x_{1}^{2}+b_{2}x_{2}^{2}+b_{3}x_{3}^{2}+b_{12}x_{1}\times x_{2}+b_{13}x_{1}\times x_{3}+\;b_{23}x_{2}\times x_{3}+b_{123}x_{1}\times x_{2}\times x_{3}+\varepsilon_{i}$$

where *y_i_* is the dielectric constant or loss factor, $b_{0},b_{i},b_{j},b_{k},b_{ii,}b_{ij},-b_{ijk},$ are the parameters, $x_{i}$ is an independent variable, such as frequency, temperature, or moisture content, and $\varepsilon_{i}$ is a model random error.

The nonstandard conditions for a linear regression model include the model misspecification, the non-constant variance, and a non-normal distribution [31,32,33]. These conditions can be identified using modern regression analysis. The diagnostic techniques are described as follows:

#### 2.1.1. Residual Plots

The distribution between residuals and the predicted values of a model is called a residual plot. A residual plot is a graph that present the residuals on the longitudinal axis and the predicted value of the model on the horizontal axis. If the data exhibit a uniform distribution along the *y_i_* = 0 line, this regression model is an appropriate model. If there is a fixed pattern for the data distribution, this model is not suitable [32,37,38,39].

#### 2.1.2. Normality Test

A normal distribution of the data is verified using the Kolmogorov–Smirnov test. The Kolmogorov–Smirnov statistic calculates the distance between the empirical distribution function of the sample and the cumulative distribution function of the normal distribution. The criterion is a *p*-value to determine the probability of being incorrect in concluding the data [32,37,38,39].

#### 2.1.3. Constant Variance Test

A constant variance test is performed by calculating the Spearman rank correlation between the observed values of dependent variables and the absolute residual values. The statistic is used to evaluate the strength and direction of the association that exists between two variables. The *p*-value is used to determine the significance of any correlation between these variables [37,38,39].

#### 2.1.4. Transformation

If the assumption of a normal distribution and constant variance are violated, data with independent or dependent variables are transformed to stabilize the error variances and to achieve a normal distribution of the data. The ordinary forms of the transformation are logarithmic (*lny*), inverse power (1/y), and square root ($\sqrt{y}$) [31,32,37,38,39].

In this study, two forms of the combination of independent variables are used (Equation (8) and Equation (9)):

(8) $$f(T,X,f)=b_{0}+b_{1}T+b_{2}X+b_{3}f+b_{11}T^{2}+b_{22}X^{2}+b_{33}f^{2}+b_{12}T\times X+\;b_{13}T\times f+b_{23}X\times f+b_{123}T\times X\times f.$$

The term *f* is transformed into logarithmic form as ln(*f*):

(9) $$g(T,X,ln\;f)=c_{0}+c_{1}T+c_{2}X+c_{3}\ln f+c_{11}T^{2}+c_{22}X^{2}+c_{33}\ln f^{2}+c_{12}T\times X+\;c_{13}T\times \ln f+c_{23}X\times \ln f+c_{123}T\times X\times \ln f.$$

Four forms of independent variables are used: *y*, $\sqrt{y}$, *lny* and 1/*y*.

The statistical analysis involved multiple linear regression. The parameters were estimated with the use of SigmaPlot v. 14.0 (SPSS Inc., Chicago, IL, USA).

### 2.2. The Effect of the Storage Time

To determine the effect of the storage time on the dielectric properties, storage time is assumed as a variable and is incorporated into the regression model. If the relationship between a dielectric property (*y*) and the logarithmic form of the frequency presented in Equation (10) is:

(10) $$\ln(y)=d_{0}+d_{1}\ln f+d_{2}\ln f^{2}$$

then, the equation (Equation (11)) to determine the effect of the storage time on the dielectric property is:

(11) $$\ln(y)=e_{0}+e_{1}\ln f+e_{2}St+e_{11}\ln f^{2}+e_{22}St^{2}+e_{12}\ln f\times St$$

where *St* is the storage time in weeks.

The effect of the storage time is tested by validating three parameters: $e_{2},e_{22}$, and $e_{12}$. If these three parameters are invalid and are not significantly different from zero, the effect of the storage time on dielectric properties is neglected.

### 2.3. Categorical Testing

To determine the effect of categorical variables, such as treatment (e.g., unsalted or salted) [13,16], position of the sample (e.g., anterior, middle, tail, and belly) [17], moisture conditions (e.g., low, medium, and high) [15], or concentration (e.g., no salted, light salted, medium salted, and heavy salted) [19], a categorical test is used.

1. Two categories:

*z* = 0, if the observation is from level A.

*z* = 1, if the observation is from level B.

If the equation (Equation (12)) linking the independent variable and two variables is:

(12) $$y_{i}=b_{0}+b_{1}x_{1}+b_{11}x_{1}^{2}+b_{2}x_{2}+b_{22}x_{2}^{2}+b_{12}x_{1}x_{2}+\varepsilon_{i}$$

then, the equation (Equation (13)) to determine the significance of two levels of a factor is

(13) $$y_{i}=c_{0}+c_{3}z+c_{1}x+c_{13}x_{1}z+c_{11}x_{1}^{2}+c_{113}x_{1}^{2}z+c_{2}x_{2}+c_{23}x_{2}z+c_{22}x_{2}^{2}+\;c_{223}x_{2}^{2}z+c_{12}x_{1}x_{2}+c_{123}x_{1}x_{2}z+\varepsilon_{i}$$

where *c*_3_, *c*_13_, *c*_113_, *c*_23_, *c*_323_, and c_113_ are constants and *z* is the categorical variable.

The effect of a factor is determined by testing the significance of *c*_3_, *c*_13_, *c*_113_, *c*_23_, *c*_323_, and *c*_113_ values.

2. Three categories:

$z_{1}=0,z_{2}=0,$ if the observation is from level A.

$z_{1}=1,z_{2}=0,$ if the observation is from level B.

$z_{1}=0,z_{2}=1,$ if the observation is from level C.

Equation (12) pertains to factors with three levels.

The equation to determine the significance of the levels of a factor is (Equation (14)):

(14) $$y_{i}=d_{0}+d_{1}x_{1}+d_{2}x_{2}+d_{11}x_{1}^{2}+d_{22}x_{2}^{2}+d_{12}x_{1}x_{2}+d_{3}z_{1}+d_{4}z_{2}+d_{13}x_{z}z_{1}+\;d_{14}x_{1}z_{2}+d_{113}x_{1}^{2}z_{1}+d_{114}x_{1}^{2}z_{1}+d_{23}x_{2}z_{1}+d_{24}x_{2}z_{2}+d_{223}x_{2}^{2}z_{1}+d_{224}x_{2}^{2}z_{2}+\;d_{123}x_{1}x_{2}z_{1}+d_{124}x_{1}x_{2}z_{2}+\varepsilon_{i}$$

The effect of factors is determined by testing the significance of *d*_3_, *d*_4_, *d*_13_, *d*_14_, *d*_113_, *d*_114_, *d*_223_, *d*_224_, *d*_123_, and *d*_124_ values.

3. Four categories:

$z_{1}=0,z_{2}=0,z_{3}=0,$ if the observation is from level A.

$z_{1}=1$, $z_{2}=0,z_{3}=0,$ if the observation is from level B.

$z_{1}=0,z_{2}=1,z_{3}=0,$ if the observation is from level C.

$z_{1}=0,z_{2}=0,z_{3}=1,$ if the observation is from level D.

The equation to determine the significance of these factors is (Equation (15)):

(15) $$y_{i}=e_{0}+e_{1}x_{1}+e_{2}x_{2}+e_{11}x_{1}^{2}+e_{22}x_{2}^{2}+e_{12}x_{1}x_{2}+e_{3}z_{1}+e_{4}z_{2}+e_{5}z_{3}+e_{13}x_{1}z_{1}+\;e_{14}x_{1}z_{2}+e_{15}x_{1}z_{3}+e_{113}x_{1}^{2}+e_{114}x_{1}^{2}z_{2}+e_{115}x_{1}^{2}z_{3}+e_{23}x_{2}z_{1}+e_{24}x_{2}z_{2}+e_{25}x_{2}z_{3}+\;e_{223}x_{1}^{2}z_{1}+e_{224}x_{1}^{2}z_{2}+e_{225}x_{1}^{2}z_{3}+e_{123}x_{1}x_{2}z_{1}+e_{124}x_{1}x_{2}z_{2}+e_{125}x_{1}x_{2}z_{3}+\varepsilon_{i}$$

### 2.4. Criterion of the Model Comparison

In this study, the dependent variables have different forms, such as *y*, $\sqrt{y}$*, lny*, and 1/*y.* The best equation could not only be determined using the *R*^2^ value of each equation. The *R*^2^ value is calculated as [31,32] (Equation (16)):

(16) $$R^{2}=\frac{\sum {\left({\hat{y}}_{i}-\bar{y}\right)}^{2}}{\sum {\left(y_{i}-\bar{y}\right)}^{2}}$$

where ${\hat{y}}_{i}$ is the predicted value for the equation, $\bar{y}$ is the average value of the dependent variable, and $y_{i}$ is the dependent variable.

The numerical values for the dependent variable are different because of its transformation form. The *R*^2^ value for model *y* is calculated using the values of ${\hat{y}}_{i}$, $\bar{y}$, and $y_{i}$. The *R*^2^ of ln(y) is calculated using ${\hat{y}}_{i}$, $\ln\bar{y}$, and $\ln y_{i}$. It is meaningless to compare the *R*^2^ values of these models.

The error variance or error mean square *s*^2^ is used to determine the fitting agreement for several models [31,32,33]. However, the transformed response value must be transformed back to the natural variable.

The calculation of *s*^2^ is for the *y_i_* (Equation (17)):

(17) $$s^{2}=\frac{\sum {\left(y_{i}-\bar{y}\right)}^{2}}{n-p}$$

For the ln(*y*) form equation, all predicted values using regression are in the form of $\ln{\hat{y}}_{i}$. The *s*^2^ value is calculated as (Equation (18)):

(18) $$s^{2}=\frac{\sum {\left(y_{i}-Exp\left(\ln{\hat{y}}_{i}\right)\right)}^{2}}{n-p}$$

The same process is used for the 1/y form of the equation, where all predicated values for regression are $\hat{\left(1/y\right)}$, and the s^2^ value is calculated as (Equation (19)):

(19) s2=∑(yi−(1yi)−1)^2n−p

### 2.5. Literature Survey

Sixteen sets of data for dialectical properties and the experimental values for frequency, temperature, and moisture content are presented in Table 2.

## 3. Results

### 3.1. The Dielectric Equations with Three Variables

#### 3.1.1. Egg White Powder

**Figure 1:**
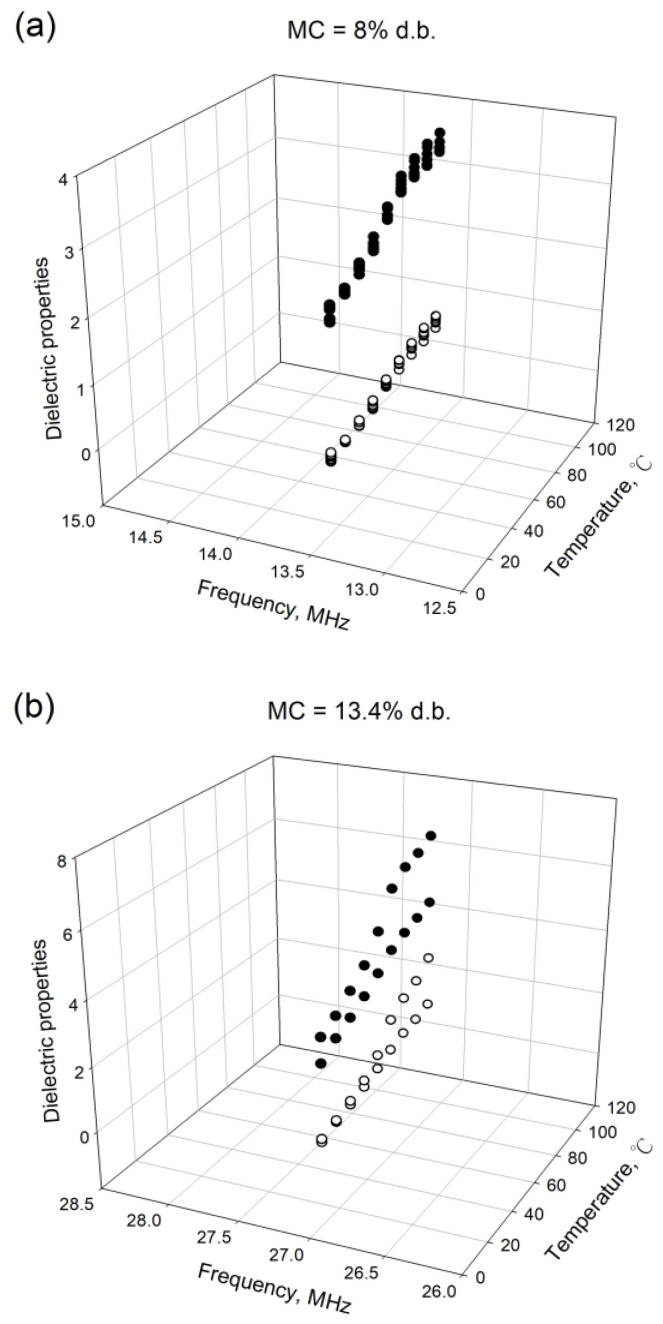
The dielectric properties of the egg white powder at 8.0% moisture content (MC) (**a**) and 13.4% d.b. moisture content (MC) (**b**); ○ is the dielectric constant; ● is the loss factor.

Two datasets are used for this literature [8]. The first dataset shows the dielectric content and loss factor data at 8.0% d.b. moisture content for five temperatures from 20 to 100 °C and five frequencies (13.56, 29.12, 40.68, 951, and 2450 MHz). The second dataset shows the dielectric properties at temperatures from 20 to 100 °C, two frequencies (27.12 and 915 MHz), and five moisture contents (5.5, 6.6, 8.0, 9.8, and 13.4% d.b.). Both datasets are pooled to derive the equations for this study. The dielectric properties of the egg white powder at 8.0% (Figure 1a) and 13.4% d.b. (Figure 1b) are showed in Figure 1.

The results for the dielectric content using modern regression analysis are listed in Table 3 (Equation (3-1) to Equation (3-8)). Four equations fulfill the criteria for the regression check. They have normally distributed data and a constant variance. The results are $\ln\left(\varepsilon^{\prime }\right)=f_{1}\left(f\right)$, $1/\varepsilon^{\prime }=f_{2}\left(f\right)$, $\ln\left(\varepsilon^{\prime }\right)=g_{1}\left(\ln f\right)$ and $1/\varepsilon^{\prime }=g_{2}\left(\ln f\right)$.

**Figure 2:**
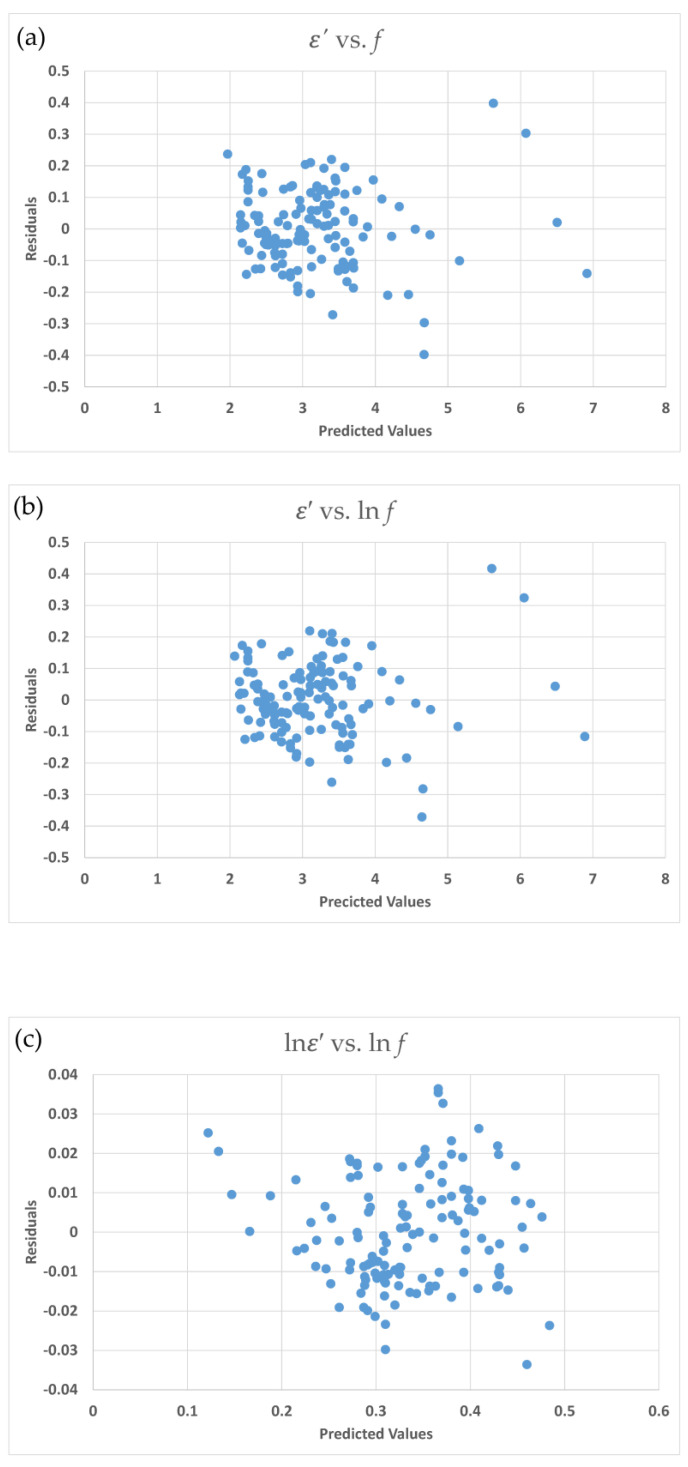

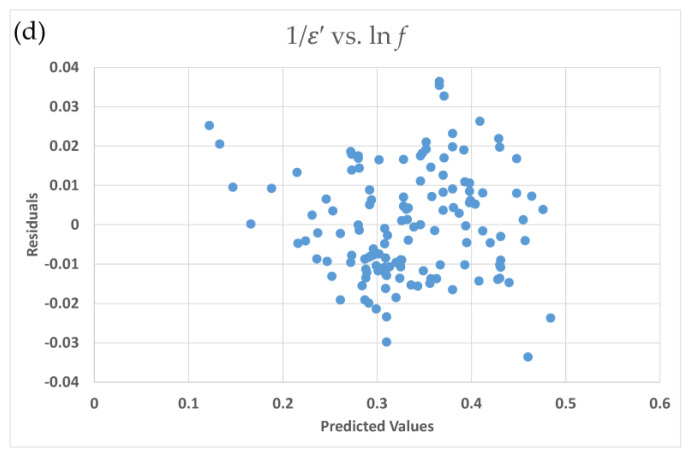
Residual plots for the dielectric constant *ε*′ equation for egg white power (Boreddy and Subbich data [8]); (**a**) $\varepsilon^{\prime }\;\mathrm{vs}.$ *f*; (**b**) $\varepsilon^{\prime }\;\mathrm{vs}.$ ln *f*, (**c**) (ln $\varepsilon^{\prime }$ vs. ln *f*); (**d**) (1/$\varepsilon^{\prime }$ vs. ln *f*).

Four residual plots are shown in Figure 2a–d. The distribution between residual and predicted values for $\varepsilon^{\prime }\;\mathrm{vs}.$ *f* (Figure 2a) and $\varepsilon^{\prime }\;\mathrm{vs}.$ ln *f* (Figure 2b) show a scatter and an inflated distribution for the residual. This demonstrates a non-constant variance for the data. The uniform distributions for residuals in Figure 2c ln $\varepsilon^{\prime }$ vs. ln *f*) and Figure 2d (1/$\varepsilon^{\prime }$ vs ln *f*) show that two equations are appropriate.

The respective s^2^ values are 0.165, 0.201, 0.125, and 0.195 for the Equation (3-3) ($\ln\left(\varepsilon^{\prime }\right)\;\mathrm{vs}.\;f_{1}\left(f\right)$, Equation (3-4) (1/ε’ vs. $f_{2}\left(f\right)$, Equation (3-7) ($\ln\left(\varepsilon^{\prime }\right)\;\mathrm{vs}.\;g_{1}(\ln f$), and Equation (3-8) ($1/\varepsilon^{\prime }\;\mathrm{vs}.\;g_{2}(\ln f$)). Equation (3-7) has the smallest s^2^ value and is the most appropriate equation for the dielectric constant.

The results of the regression analysis for the loss factor for egg white powder are listed in Table 4.

**Figure 3:**
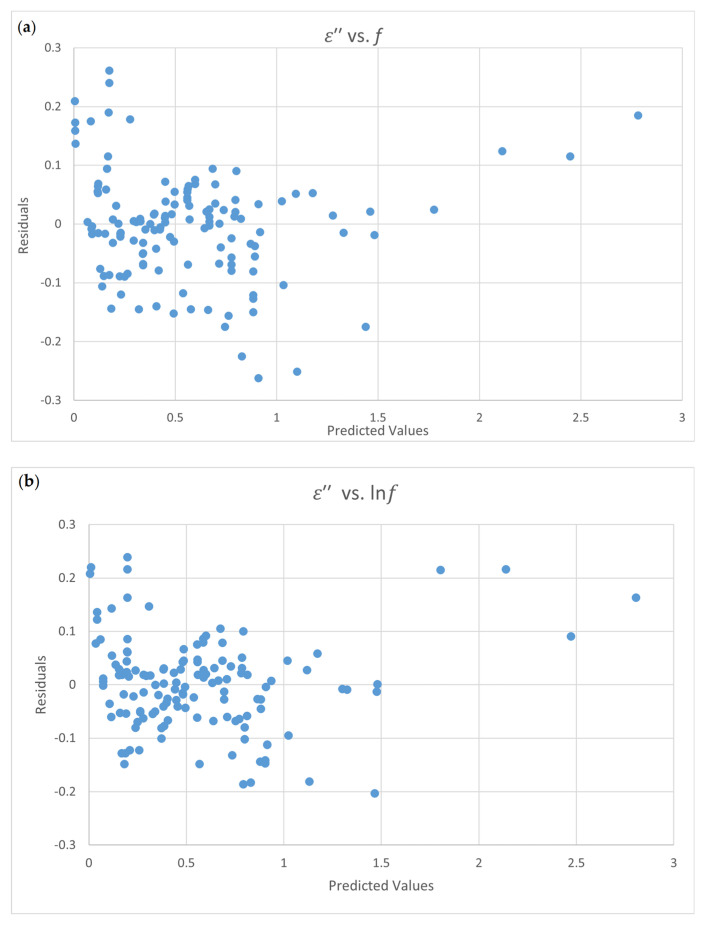
Residual plots for the loss factor ε’’ equation for egg white power (Boreddy and Subbich data [8]); (**a**). $\varepsilon^{\prime \prime }$ vs. *f*; (**b**). $\varepsilon^{\prime \prime }\;\mathrm{vs}.\;\ln f$.

Only Equation (4-5) passes the normal test and the constant variance test. The residual plots of $\varepsilon^{\prime \prime }$ vs. *f* and the $\varepsilon^{\prime \prime }\;\mathrm{vs}.\;\ln f$ are shown in Figure 3. Figure 3a shows the scatter data distribution and the scatter conditions for non-constant variance. The uniform distribution of data in Figure 3b presents that this equation is appropriate in terms of a *t*-test for each coefficient. The terms $X^{2}$ and $\ln f^{2}$ have no significant effect on the loss factor. The final equation for the relationship between the loss factor and the influencing factors is shown in Equation (4-9).

The distribution of residual plots are lookalike for Figure 3a,b. However, the results of the normality test and the constant variance test are different. The adequateness of the model fitting cannot be verified only with visual methods of residual plots.

In the literature of the dielectric properties of egg white powder [8], the multiple regression variables were moisture constant and temperature. Each frequency had a specific equation. For our study, frequency is a variable, and only two equations (dielectric constant and loss factor) are required.

#### 3.1.2. Chicken Flour

The dielectric properties of chicken flour are determined for moisture contents from 7.8% to 20.9% w.b., temperature from 20 to 90 °C, and frequencies of 27, 40, 100, 915, and 1800 MHz [11]. No regression models are presented in the literature [11].

The relationship between dielectric constant and the influencing factors (moisture content, temperature, and frequency) was established using regression analysis, and the results are listed in Supplementary Materials Tables S1 and S2.

The adequate equations are listed as the following (Equation (20) and Equation (21)):

1/*ε*′ = 0.560 − (0.00263 × *T*) + (0.0000684 × *f*) − (0.0161 × *X*) − (0.0000110 × *T*^2^) − (0.0000000260 × *f*^2^) + (0.000117 × *X*^2^) + (0.000000459 × *T* × *f*) + (0.00000309 × *T* × *X*) + (0.00000239 × *f* × *X*) − (0.0000000544 × *T* × *f* × *X*) (20)

ln(*ε*″) = 3.935 − (0.268 × *T*) − (0.339 × *X*) − (0.595 × ln *f*) + (0.00300 × *T*^2^) + (0.0584 × ln *f*^2^) + (0.00903 × *X*^2^) + (0.00888 × *T* × ln *f*) + (0.0122 × *T* × *X*) − (0.000623 × *T* × ln *f* × *X*) − (0.0000115 × *T^3^*) − (0.00000589 × (*T* × *f*) ^2^) − (0.00000191 × (*T* × X)^2^). (21)

**Figure 4:**
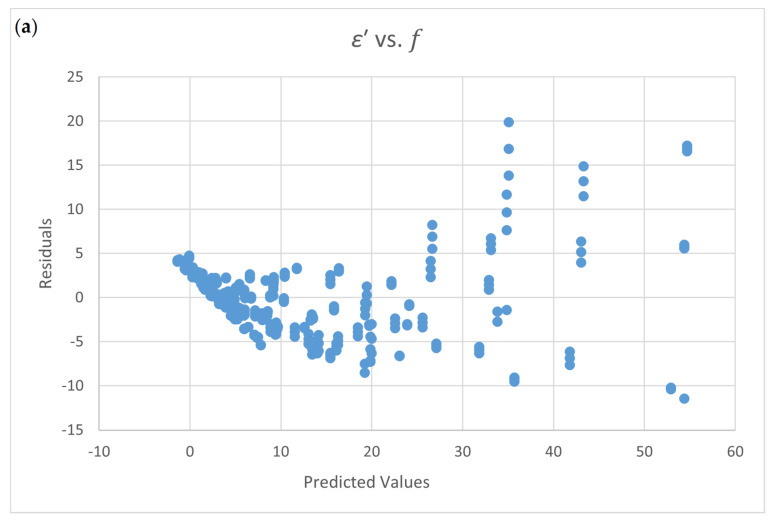

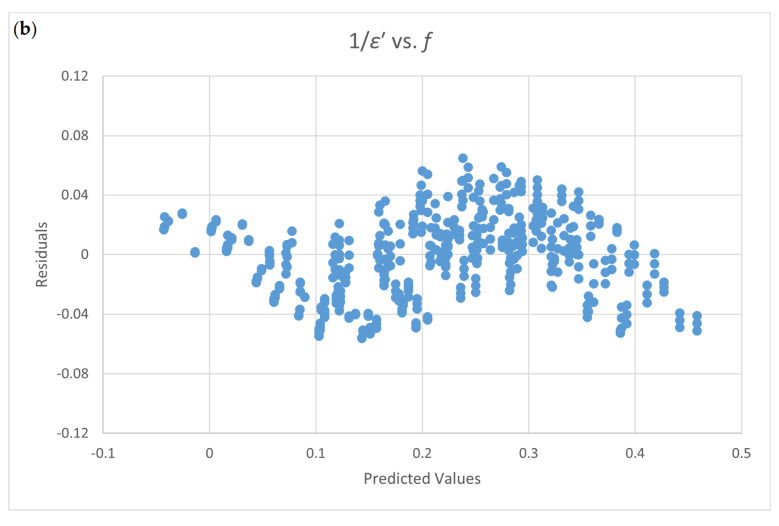
Residual plots for the dielectric constant ε’ equation for chicken flour (Guo et al. data [11]); (**a**). *ε*′ vs. f; (**b**). 1/*ε*′ vs. *f.*

In Supplementary Materials Table S1, the only adequate equation, 1*/ε*′, is a dependent variable, and moisture content, temperature, and frequency are independent variables (Equation (20)). Other models cannot pass the normal test or the constant variance test. The residual plots for *ε*′ vs. f and 1/*ε*′ vs. *f* show the data distribution of errors. Figure 4a shows a funnel-type data distribution and a non-constant variance error. Figure 4b shows a uniform distribution for predicted errors.

**Figure 5:**
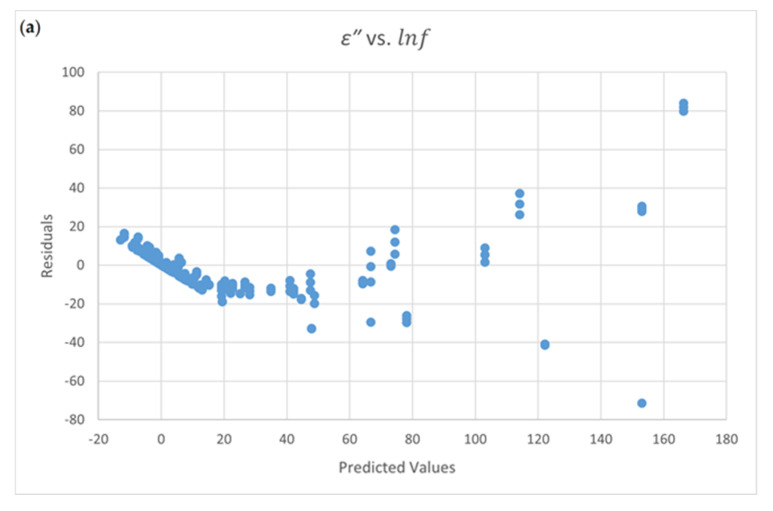

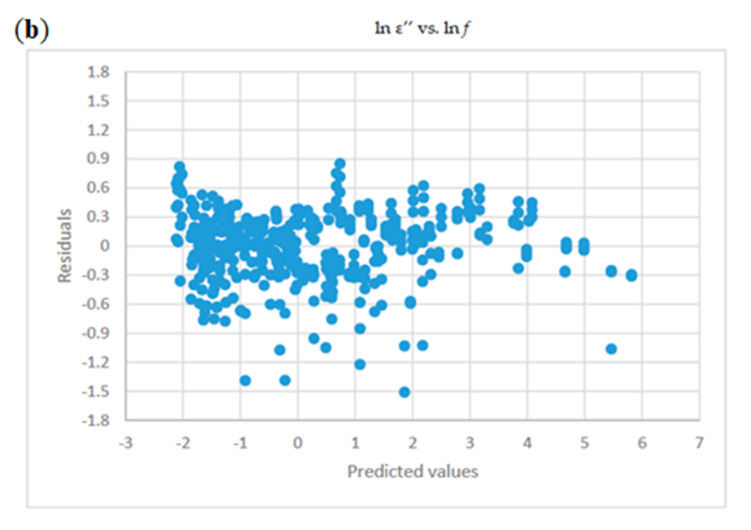
Residual plots for the loss factor ε’’ equation for chicken flour (Guo et al. data [11]); (**a**). *ε*″ vs. lnf; (**b**) $\ln\;\varepsilon^{\prime \prime }\mathrm{vs}.\;\ln f$.

Only the equation, $\ln\left(\varepsilon^{\prime \prime }\right)\mathrm{vs}.\;g\left(X,\;T,\ln f\right)$, is an appropriate model. These variables have a more complex form, e.g., *T^3^*, (*T ×* ln *f*)^2^ and (*T × X*)^2^. The residual plots for *ε*″ vs. lnf are shown in Figure 5a. The funnel effect shows a heterogeneous variance. In Figure 5b, the $\ln\varepsilon^{\prime \prime }\mathrm{vs}.\;\ln f$ model has a uniform distribution for residual data.

**Figure 6:**
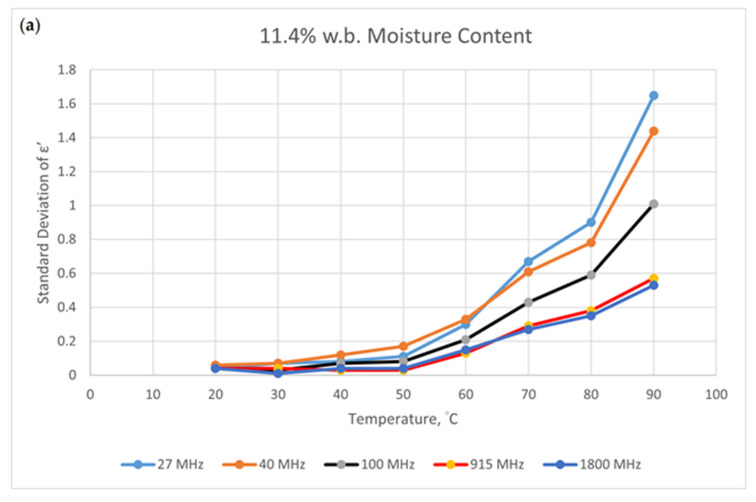

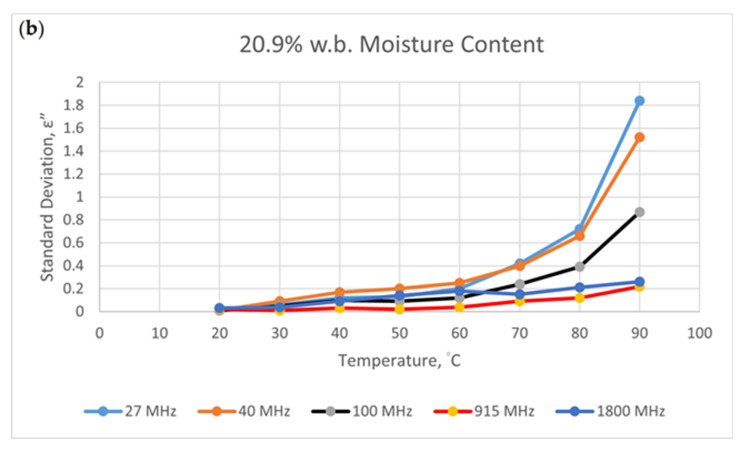
The relationship between the standard deviation of the measured data and the temperature at different frequencies for two moisture contents; (**a**). 11.4% w.b. moisture content; (**b**). 11.4% w.b. moisture content.

There is a heterogeneous variance for dielectric properties in the standard deviations for each measurement [11]. The standard deviation is calculated using three sets of measurements. Two typical data distributions are shown in Figure 6. Figure 6a shows the standard deviations for the dielectric constant for different temperatures and frequencies at 11.4% w.b. The standard deviation increases as temperature increases. The lower the frequency, the greater the standard deviation. The numerical values for 915 and 1800 MHz are similar.

The standard deviation of the loss factor at 20.9% w.b. is shown in Figure 6b. The greatest value is for 80 °C. Lower frequencies have greater standard deviations. The non-even values of standard deviations show the origin of the heterogeneous variance.

#### 3.1.3. Bread

The dielectric properties for white bread with different moisture contents (34.0, 34.6, 37.1, and 38.6% w.b.) were determined at frequencies of 13.56, 27.12, 40.68, 915, and 1800 MHz and at temperature of 25 to 85 °C [14]. The results for regression are listed in Supplementary Materials Table S3. The relationship between response 1/ε′ and the three variables (*X*, *T*, ln *f*) is the only appropriate equation for the dielectric constant (Supplementary Materials Table S3). Further analysis indicates that *T*^2^ and ln *f*^2^ do not have a significant effect on response. The best equation is listed as follows (Equation (22)):

1/*ε*′ = 1.236 − (0.0136 × *T*) − (0.0289 × *X*) + (0.0959 × ln *f*) − (0.00626 × ln *f*^2^) + (0.000194 × *T* × ln *f*) + (0.000307 × *T* × *X*). (22)

The relationship between $\ln\left(\varepsilon^{\prime \prime }\right)$ and *X*, *T* and ln *f* is the only model that passes the normal test and the constant variable test (Equation (23)).

ln(*ε*″) = −8.366 + (0.136 × *T*) + (0.336 × *X*) − (0.716 × ln *f*) + (0.0000507 × *T*^2^) + (0.0793 × ln *f*^2^) − (0.00369 × *T* × ln *f*) − (0.00291 × *T* × *X*) − (0.0137 × ln *f* × *X*) (23)

The literature of the dielectric properties of bread [11] uses three equations. For 37.1% w.b. and five temperatures, the *ε*′ and ε″ response is described as *b*_0_ *+ b*_1_*/f.* At a fixed temperature of 25 °C and five frequencies, the dielectric properties have a linear relationship with moisture constant: $c_{0}+c_{1}X$. At a fixed frequency of 27.12 MHz and four moisture contents, the relationship between the dielectric properties and temperature is a 2nd order polynomial equation: $d_{0}+d_{1}T+d_{2}T^{2}$.

In our study, the independent variables are influencing factors (moisture content, temperature, and frequency) and are incorporated into a multiple regression equation. This is a more useful and convenient method to derive the equations for the dielectric properties of bread.

#### 3.1.4. Black-Eyed Peas

The predicted equations for dielectric properties of black-eyed peas were studied using regression analysis (Table 5). The dataset from the literature [12] applies to four moisture contents (8.8, 12.7, 16.8, and 20.3% w.b.), three frequencies (27, 40, and 915 MHz) and five temperatures (20–60 °C). For the dielectric content, two equations (Equations (5-1) and (5-2)) pass the normal test and the constant variance test.

The transformations of the responses are $\ln\varepsilon^{\prime }$ and $1/\varepsilon^{\prime }$. A comparison of the error mean square for two models shows that the fitting-agreement for $\ln\varepsilon^{\prime }$ is better than that for 1/$\varepsilon^{\prime }$.

In terms of the loss factor, lnε″ vs. g (T,X,lnf) is the only appropriate equation and is listed as shown in Equation (5-3).

#### 3.1.5. Macadamia Nut Kernels

The dielectric properties of macadamia nut kernels were determined at five temperatures, five moisture contents (%, w.b.), and four frequencies (27, 40, 915, and 1800 MHz) [18]. The literature did not cite related empirical equations.

The results of the regression analysis are listed in Table 6. The best equations for dielectric proportion are Equations (6-1) and (6-2). In terms of the $\varepsilon^{\prime }$ and $\varepsilon^{\prime \prime }$ response, $\ln\varepsilon^{\prime }\mathrm{or}$ $\ln\varepsilon^{\prime \prime }$ vs. g(T,X,lnf) are the only equations that pass the normal test and the non-constant variance test.

### 3.2. Equation for Dielectric Properties with Two Variables

Some experiments consider only temperature and frequency as the influencing factors. The moisture content of fruits, vegetables, and insects was not measured because their moisture content is very high.

#### 3.2.1. Liquid and Precooked Egg White

This literature used two types of egg whites: liquid and pre-cooked. The dielectric properties were determined at four frequencies (27, 40, 915, and 1800 MHz) and seven temperatures (20–120 °C, at intervals of 20 °C).

The results of the regression analysis are listed in Supplementary Materials Table S5. For the dielectric constants for liquid egg white, the transformation of $\ln\varepsilon^{\prime }$ and 1/$\varepsilon^{\prime }$ passes the normal test and the constant variance test (Equations (24) and (25)). A comparison of the error mean square shows that the two *s*^2^ values are similar, so both equations are useful models. In terms of the loss factors, the transformation of $\ln\varepsilon^{\prime \prime }$ (Equation (26)) is the only equation that expresses the relationship between the $\varepsilon^{\prime \prime }$ value and temperature and moisture content for liquid egg white.

The adequate equations are listed as follows (Equations (24)–(26)):

ln(*ε*′) = 5.736 + (0.000700 × *T*) − (0.548 × ln *f*) + (0.0000632 × *T*^2^) + (0.0490 × ln *f*^2^) − (0.00155 × *T* × ln *f*) (24)

*1/ε*′ = −0.00480 + (0.0000107 × *T*) + (0.00662 × ln *f*) − (0.000000872 × *T*^2^) − (0.000565 × ln *f*^2^) + (0.0000193 × *T* × ln *f*) (25)

ln(*ε*″) = 9.793 + (0.0169 × *T*) − (1.403 × ln *f*) − (0.00000389 × *T*^2^) + (0.0553 × ln *f*^2^) − (0.00113 × *T* × ln *f*) (26)

For pre-cooked egg white, the results for appropriate models are listed in Supplementary Materials Table S5. The transformation form of $\ln\varepsilon^{\prime \prime }$ and 1/$\varepsilon^{\prime \prime }$ is used for the dielectric constant (Equation (27)) and the loss factor (Equation (28)).

ln(*ε*′) = 5.552 + (0.00355 × *T*) − (0.443 × ln *f*) + (0.0000269 × *T*^2^) + (0.0372 × ln *f*^2^) − (0.00140 × *T* × ln f) (27)

1/*ε*″ = 0.0525 + (0.000245 × *T*) − (0.0292 × ln *f*) + (0.000000199 × *T*^2^) + (0.00438 × ln *f*^2^) − (0.0000852 × *T* × ln *f*) (28)

In this literature, the relationship between dielectric properties and temperature at a fixed frequency is expressed as a 2nd-order polynomial equation ($y=b_{0}+b_{1}T+b_{2}T^{2}$). The coefficient of determination $R^{2}$ is the only criterion. There are sixteen equations for the dielectric constant and the loss factor in this literature. For our study, the temperature and frequency are used, and the significant effect of the interaction between temperature and moisture (*X* × *T* in the equation) is determined.

#### 3.2.2. Fruits, Nuts, and Insects

In order to develop, improve, and scale up the electromagnetic treatment devices for insect pests, basic information about dielectric properties was collected [20]. Wang et al. [20] studied the dielectric properties of five types of fruits, two types of nuts, and four insect larvae at four frequencies (27, 40, 915, and 1800 MHz) and five temperatures (20–60 °C).

The best equation was tested using regression analysis. The results are listed in Table 7. Most of the products only have an equation that passes the statistical test for normal distribution and constant variance. For the dielectric constant of Gold apple, two transformations of the response, $\ln\varepsilon^{\prime }$ and 1/$\varepsilon^{\prime }$, are used. For the dielectric properties of cherry and orange, four equations are adequate: ($\varepsilon^{\prime },\sqrt{\varepsilon^{\prime }},\;\ln\varepsilon^{\prime },1/\varepsilon^{\prime }$).

For the dielectric constant of walnut, $\varepsilon^{\prime }$ and $\sqrt{\varepsilon^{\prime }}$ are used to establish an equation. For the dielectric constant of Indian-meal moth, $\sqrt{\varepsilon^{\prime }}$ and $ln\varepsilon^{\prime }$ are the appropriate form for the equation. Only one equation is used for other products, and the appropriate equations are listed in Table 7.

For the loss factor for grapefruit, $ln\varepsilon^{\prime },1/\varepsilon^{\prime }$ is used. For the loss factor for walnut, $\sqrt{\varepsilon^{\prime }}$ and $ln\varepsilon^{\prime }$ are the appropriate form for the prediction equation. For other products, only one equation is appropriate. These equations are listed in Table 7.

### 3.3. Effect of the Storage Time on the Dielectric Properties of Eggs

This literature showed the dielectric properties of egg albumen and egg yolk at six frequencies (10, 27, 40, 100, 915, and 1800 MHz) over five weeks (0, 1, 2, 3, 4, and 5 Ws). In this literature, an analysis of variance (ANOVA) was used to determine the significance of the storage time on the dielectric properties at each frequency. However, the statistical test for this literature does not consider the effect of the frequency on the dielectric properties.

Regression analysis was applied to determine the relationship between dielectric properties and frequency. The storage time is assumed to be an influencing factor, and the regression equation involving storage time was tested. For the dielectric properties of egg yolk and albumen, the best equation has the form $\ln y=b_{0}+b_{1}\ln f+b_{2}{\left(\ln f\right)}^{2}$. The equation for the effect of the storage time is $\ln\left(y\right)=c_{0}+c_{1}St+c_{2}\ln f+c_{11}St^{2}+c_{22}{\left(\ln f\right)}^{2}+c_{12}St\times (\ln f$). The results are presented in Table 8.

The results of regression equations are shown as Equations (8-2), (8-4), (8-6), and (8-8). The results of a *t*-test for each of the parameters shows that the variables, *St*, *St*^2^ and (lnf)×St, are not significantly different to zero, so the term for the storage time is omitted from these equations. The results of this regression test show that the frequency has a significant effect on the dielectric properties. However, the storage time has no significant effect.

### 3.4. Categorical Test of Two Factors

#### 3.4.1. Butter

The dielectric properties of butter were tested at two frequencies (915 and 2450 MHz) and six temperatures (30–80 °C) for two treatments: salted and unsalted in the literature [13]. The equations in the literature showed the relationships between the dielectric properties and the temperature at a fixed frequency and for different treatments. The significance of salt levels on dielectric properties was determined by visually verifying from the data distribution in the figures.

In our study, the effect of the salted treatment is an indicating factor in the regression equation. The results are listed in Table 9. The categorical factor (salted treatment) is denoted as *z*. Three *z* terms, $T^{2}\times z$, and lnf×z were validated using a *t*-test for each coefficient. It was found that salted treatment has a significant effect on the dielectric content and the interaction between $T^{2}$ and the $\ln f^{2}$ term.

The dielectric constant equation includes the categories (Equations (29)–(31)):

ln(ε′) = 3.777 + (1.370 × *z*) + (0.00355 × *T*) + (0.00433 × *T* × *z*) − (0.000160 × *T*^2^) + (0.000151 × *T*^2^ × *z*) − (0.0680 × ln *f*) − (0.349 × ln *f* × *z*) (29)

For unsalted, z = 0, so the dielectric constant equation is

ln(*ε*′) = 3.777 + (0.00355 × *T*) − (0.000160 × *T*^2^) − (0.0680 × ln *f*) (30)

For salted, z = 1, so the dielectric constant equation is (Equation (31)):

ln(*ε*′) = 5.147 + (0.00788 × *T*) − (0.000009 × *T*^2^) − (0.417 × ln *f*) (31)

The effect of the salted butter on the loss factor is described by Equation (13-2). The terms *z*, T×z, and ln f×z are valid for this equation. Salted treatment has to have a significant effect on the loss factor for butter.

The loss factor equation includes the categories (Equations (32)–(34)):

ln *ε*″ = 3.978 + (19.066 × *z*) − (0.0126 × *T*) + (0.0902 × *T* × *z*) − (0.00000579 × *T*^2^) − (0.000241 × *T*^2^ × *z*) − (0.192 × ln *f*) − (2.600 × ln *f* × *z*) (32)

For unsalted, z = 0, so the loss factor equation is

ln *ε*″ = 3.978 − (0.0126 × *T*) − (0.00000579 × *T*^2^) − (0.192 × ln *f*) (33)

For salted treatment, z = 1, so the loss factor equation is

ln *ε*″ = 23.044 − (0.0776 × *T*) − (0.000208 × *T*^2^) − (2.792 × ln *f*) (34)

The different equations for two treatments showed the significant effect of the salted treatment on the loss factor.

#### 3.4.2. Salmon Fish

The dielectric properties of salmon (*Oncorhynchus keta*) at two frequencies (27 and 915 MHz) and seven temperatures (20–80 °C) and two types of treatments (unsalted and salted) were reported [16]. The effect of salting treatment on the dielectric properties of salmon were observed visually, and the data distribution for measured values is presented in the figures. At 27 MHz, the dielectric properties for salted salmon were higher than those for unsalted salmon, but at 915 MHz, the two datasets (salted and unsalted) were very difficult to observe visually.

The results for the categorical test of dielectric properties of salmon are listed in Table 9. For the dielectric constant (Equation (9-3)), the terms, *z*, T×z, and lnf×z, are validated using a *t*-test. Salted treatment has a significant effect on the dielectric constant.

The result of the categorical test for loss factors (Equation (9-4)) shows that the three terms, *z*, T×z*,* and lnf×z, are valid. Salted treatment has a significant effect on these loss factors.

The regression equation involves categorical factors, and the effect of the salted treatment can be quantified.

### 3.5. Categorical Test of Three Factors

The dielectric properties of cheese were tested for a frequency range of 300 to 3000 MHz at temperatures between 5 and 85 °C in intervals of 10 °C [15]. There are three moisture levels for the test materials: low, medium, and high. The effect of the moisture content on these properties is shown in the figures in the literature [15]. The data distribution for dielectric properties for three levels of the moisture content was observed visually, but it is difficult to ascertain significant patterns in this data.

The levels of the moisture content in samples are categorical factors, and the regression results are listed in Table 10. The form of the dielectric constant is $\sqrt{\varepsilon^{\prime }}$. The categorical terms $z_{1}$, $T\times z_{1}$, and $T^{2}\times z_{1}$, are valid, but the variables, $z_{2}$, $T\times z_{2}$, and $T^{2}\times z_{2},$ are invalid. The dielectric constant for low moisture content is significantly different to those for medium and high moisture content.

The result of the regression equation for the loss factor is calculated using Equation (10-2). The categorical variables, $z_{1},z_{2}$, and other interaction terms are valid. The result indicates that three moisture levels have a significant effect on the loss factor for cheese.

### 3.6. Categorical Test of Four Factors

#### 3.6.1. Salmon Fillets

Four positions of dielectric properties of Alaska pink salmon fillets (anterior, middle, tail, and belly) were tested at five frequencies (27, 40, 433, 915, and 1800 MHz) and six temperatures (from 20 to 120 °C in intervals of 20 °C). The dielectric properties of salmon fillets at four positions at different frequencies and temperatures were observed in the literature [17]. In our study, the effect of the position of salmon on the dielectric properties was tested using the categorical method, and the results of regression analysis are listed in Table 11.

#### 3.6.2. Pecan Kernels

The dielectric properties of pecan kernels with four levels of salted contents (none, light, medium, and heavy) were determined at 15% w.b. moisture content, at four temperatures and four frequencies.

The effect of salted levels on the dielectric properties was determined using a categorical test. The results are shown in Table 12.

For the dielectric constant, the variables of three categorical factors ($z_{1},z_{2},\;\mathrm{and}\;z_{3}$) and their interaction with temperature and frequency are valid (Equation (12-1)). Therefore, salted levels have a significant effect on the dielectric constant of pecan nuts at 15% w.b. MC (moisture content).

Equation (12-2) shows the results of the categorical test on loss factors. All variables involving categorical factors and their interaction variables are valid. The results show that the salted levels have a significant effect on the loss factor of pecan kernels at 15% w.b. moisture content.

In our study, the effect of salted levels was quantified using categorical factors. The regression equations for the dielectric properties for different salted levels at 15% w.b. moisture content are easily derived.

For the dielectric constant, the terms of categorical variables *z*_1_, *z*_2_, *T* × *z*_3_, ln *f* × *z*_1_, ln *f* × *z*_2_, and ln *f* × *z*_3_ were validated using a *t*-test, so the position of salmon fillets has a significant effect on the dielectric constant.

The effect of the position of salmon fillets on the loss factor was determined, and the regression result is presented using Equation (11-2). All terms for categorical variables (*z*1, *z*2, *T × z*3, ln *f × z*1, ln *f × z*2 and ln *f × z*3, etc.) are invalid. There is no significant difference between these datasets for loss factors for different positions of salmon.

### 3.7. The Best Regression Equations for Each Food Ingredient

From the results of this study, the best regression equations (Equations (35)–(52)) for each food ingredient are listed as follows:

#### 3.7.1. Egg White Powder

ln(*ε*′) = 0.400 − (0.00242 × *T*) + (0.0434 × ln *f* + (0.0140 × *X*) − (0.0000462 × *T*^2^) − (0.00271 × ln *f*^2^) + (0.00180 × *X*^2^) + (0.00181 × *T* × ln *f*+ (0.00159 × *T* × *X*) − (0.00422 × ln *f* × *X*) − (0.000197 × *T* × ln *f* × *X*) (35)

ε″ = 1.975 − (0.0471 × *T*) − (0.347 × *X*) − (0.178 × ln *f*) + (0.00932 × *X*^2^) + (0.00723 × *T* × ln *f*) + (0.00729 × *T* × *X*) + (0.0236 × ln *f* × *X*) − (0.000924 × *T* × ln *f* × *X*) (36)

#### 3.7.2. Chicken Flour

1/*ε*′ = 0.560 − (0.00263 × *T*) + (0.0000684 × *f*) − (0.0161 × *X*) − (0.0000110 × *T*^2^) − (0.0000000260 × *f*^2^) + (0.000117 × *X*^2^) + (0.000000459 × *T* × *f*) + (0.00000309 × *T* × *X*) + (0.00000239 × *f* × *X*) − (0.0000000544 × *T* × *f* × *X*) (37)

ln(*ε*″) = 3.935 − (0.268 × *T*) − (0.339 × *X*) − (0.595 × ln *f*) + (0.00300 × *T*^2^) + (0.0584 × ln *f*^2^) + (0.00903 × *X*^2^) + (0.00888 × *T* × ln *f*) + (0.0122 × *T* × *X*) − (0.000623 × *T* × ln *f* × *X*) − (0.0000115 × *T^3^*) − (0.00000589 × (*T* × *f*) ^2^) − (0.00000191 × (*T* × *X*)^2^) (38)

#### 3.7.3. Bread

1/*ε*′ = 1.236 − (0.0136 × *T*) − (0.0289 × *X*) + (0.0959 × ln *f*) − (0.00626 × ln *f*^2^) + (0.000194 × *T* × ln *f*) + (0.000307 × *T* × *X*) (39)

ln(*ε*″) = -8.366 + (0.136 × *T*) + (0.336 × *X*) − (0.716 × ln *f*) + (0.0000507 × *T*^2^) + (0.0793 × ln *f*^2^) − (0.00369 × *T* × ln *f*) − (0.00291 × *T* × *X*) − (0.0137 × ln *f* × *X*) (40)

#### 3.7.4. Black-Eyed Peas

ln(*ε*′) = 2.521 − (0.0201 × *T*) − (0.195 × *X*) + (0.0184 × ln *f*) + (0.000242 × *T*^2^) − (0.000327 × ln *f*^2^) + (0.00926 × *X*^2^) + (0.000677 × *T* × ln *f*) + (0.000693 × *T* × *X*) − (0.00662 × ln *f* × *X*) − (0.0000247 × *T* × ln *f* × *X*) − (0.00000124 × (*T* × *X*)^2^) (41)

ln(*ε*″) = 4.531 − (0.0719 × *T*) − (0.375 × *X*) − (1.349 × ln *f*) + (0.000639 × *T*^2^) + (0.0909 × ln *f*^2^) + (0.0131 × *X*^2^) + (0.00674 × *T* × ln *f*) + (0.00315 × *T* × *X*) + (0.0161 × ln *f* × *X*) − (0.0000621 × *T* × ln *f* × *X*) − (0.00000770 × *T* × ln *f*^2^) − (0.000000970 × (*T* × *X*)^2^) (42)

#### 3.7.5. Macadamia Nut Kernels

ln(*ε*′) = 1.467 − (0.000659 × *T*) + (0.102 × *X*) − (0.119 × ln *f*) + (0.0000327 × *T*^2^) + (0.0170 × ln *f*^2^) + (0.00000963 × *X*^2^) − (0.000227 × *T* × ln *f*) + (0.000304 × *T* × *X*) − (0.00904 × ln *f* × *X*) − (0.00000325 × *T* × ln *f* × *X*) − (0.000000205 × *T* × ln *f*^2^) − (0.0000000679 × (*T* × *X*)^2^) (43)

ln(*ε*″) = -1.719 + (0.00660 × *T*) + (0.469 × *X*) − (0.351 × ln *f*) + (0.0000602 × *T*^2^) + (0.0627 × ln *f*^2^) − (0.00381 × *X*^2^) − (0.00102 × *T* × ln *f*) + (0.000778 × *T* × *X*) − (0.0397 × ln *f*) × *X*) − (0.000118 × *T* × ln *f* × *X*) + (0.000000144 × (*T* × *X*)^2^) (44)

#### 3.7.6. Liquid Egg White

ln(*ε*′) = 5.736 + (0.000700 × *T*) − (0.548 × ln *f*) + (0.0000632 × *T*^2^) + (0.0490 × ln *f*^2^) − (0.00155 × *T* × ln *f*) (45)

ln(*ε*″) = 9.793 + (0.0169 × *T*) − (1.403 × ln *f*) − (0.00000389 × *T*^2^) + (0.0553 × ln *f*^2^) − (0.00113 × *T* × ln *f*) (46)

#### 3.7.7. Precooked Egg White

ln(*ε*′) = 5.552 + (0.00355 × *T*) − (0.443 × ln *f*) + (0.0000269 × *T*^2^) + (0.0372 × ln *f*^2^) − (0.00140 × *T* × ln *f*) (47)

1/*ε*″ = 0.0525 + (0.000245 × *T*) − (0.0292 × ln *f*) + (0.000000199 × *T*^2^) + (0.00438 × ln *f*^2^) − (0.0000852 × *T* × ln *f*) (48)

#### 3.7.8. Almond

(49) $$\sqrt{\varepsilon^{\prime }}=5.816-(0.00407\times T)-(1.357\times \ln\;f)+(0.0000683\times T^{2})+(0.112\times \ln\;f^{2})$$

(50) $$\sqrt{\varepsilon \prime \prime }=-6.515-(0.0200\times T)+(3.221\times \ln\;f)+(0.000132\times T^{2})-(0.278\times \ln\;f^{2})+(0.00144\times T\times \ln\;f)$$

#### 3.7.9. Walnut

*ε*′ = 11.067 − (0.0193 × *T*) − (2.308 × ln *f*) + (0.132 × ln *f*^2^) + (0.00855 × *T* × ln *f*) (51)

ln(*ε*″) = -7.867 − (0.0248 × *T*) + (3.187 × ln *f*) + (0.000201 × *T*^2^) − (0.264 × ln *f*^2^) − (0.000704 × *T* × ln *f*) (52)

By inspecting these best equations, no universal equation could be used to express the relationship between dielectric properties and influencing factors. Each food ingredient has its special appropriate prediction equation.

## 4. Discussion

The dielectric properties of foods are necessary elements of food technology and engineering. The factors that affect dielectric properties include quantitative and qualitative variables. The applied frequency, ambient temperature, bulk density, and moisture content of samples are quantitative variables. The treatment method, position of samples, and constituents of foods are qualitative variables.

A modern regression analysis allows the prediction equations of dielectric properties to be established, and the quantitative factors are the dependent variables. The interaction term and square term for variables is integrated into these equations. The basic assumption is that there is a normal distribution and a constant variance in data. The qualitative factors, such as salted or unsalted and position of samples, are determined using categorical testing.

This study uses the quantitative factors, such as moisture content, frequency, and temperature, to establish the modern regression analysis. To obtain the appropriate equations for the dielectric properties, the dielectric constant and loss factor for dependent variables and the frequency of independent variables is sometimes transformed as a logarithmic value (*lny*), an inverse power (1/y), or a square root ($\sqrt{y}$). Then, adequate equations of the dielectric constant and loss factor are established using modern regression analysis.

In this study, two bases of the moisture content, percent dry basis, M_db_ [8], and percent wet basis, M_wb_ [11,12,14,18,19], were used to express the moisture content of products. After checking the fitting agreement of the regression analysis models, dry or wet bases all could be used to establish the prediction equations. Both moisture bases easily convert with the equation M_wb_ = M_db/_(100 + M_db_).

For other studies, the four quantitative factors are frequency, moisture content, temperature, and density [26]. To develop a moisture meter, three quantitative factors are involved: moisture content, frequency, and the bulk density of grains and seeds [40]. A modern regression analysis can be used to establish the equations for the dielectric properties using these datasets.

A regression equation defines which dependent variables (influencing factors) are important. These equations can be used for prediction in the development of heating equipment that uses electromagnetic wave energy. Most of the appropriate equations are the form of ln(*y*) or 1/*y* for dependent variables and temperature, moisture, logarithmic form of the frequency, and their power and interaction terms for independent variables. The best form of the best equation is $c_{0}+c_{1}T+c_{2}X+c_{3}\ln f+c_{11}T^{2}+c_{22}X^{2}+c_{33}Lnf^{2}+c_{12}T\times X+c_{13}T\times lnf+c_{23}X\times lnf$.

All the published data for the dielectric properties in the literature that are listed in Table 2 are measured with an impedance analyzer with the open-end coaxial-line probe. This method was introduced and detailed [3,4,6]. This method is particularly suitable for food materials of liquid and semi-solids. The advantages of this method are simple to use and there is no damage for the sample [3,4,5,6]. The reports of the accuracy for this method is ±5% and it could be improved to ±2% after careful calibration [41]. All the literature related to the published data in Table 2 mention the calibration procedure. However, the accuracy of the dielectric properties of foods was not reported. The effect of the measurement errors on the regression analysis equations needs to be studied further. The method to calculate the measurement uncertainty on the prediction equation could be adopted [42].

In this study, six categories of foods were studied: eggs, vegetables, dairy products, fishes, nuts, and insects. The application of dielectric properties on the food processing includes measuring, heating, and classification. The moisture content and water activity of foods can be determined by the design and calibration of electrical instruments. The moisture content and water activity of foods can be measured by detecting the dielectric constant of foods and then be calculated with previous established calibration equations. The dielectric properties provide the basic information to the construction of heating devices with microwave frequencies. The disinfection of insect pests in foods could be performed by heating. The dielectric properties of fruits, nuts, and insect pests support the requirement information to arrange with appropriate frequency and time [20]. The effect of the treatment such as salted and unsalted has a significant effect on the dielectric properties of foods. The measurement of dielectric properties proposes a possible way to quantify the treatment of foods [15,16,17,19].

The power density of the thermal energy of foods can be expressed as shown in Equation (2). The relationship between loss factor $\varepsilon^{\prime \prime }$ can be predicted with the established equation of this study. That is, the power density could be computed in the specific conditions of the moisture content, temperature, and frequency. When the prediction equations of loss factors of insects and nuts or fruits are established, the disinfection system of insect pests can be design to control insects without damaging food products. With the measurement of dielectric properties in the preset frequency and temperature, the moisture content of food materials can be calculated by prediction equations. The significant effect of the dielectric properties of foods with different treatments could be evaluated by the adequate predicted equations of products and categorical testing of the regression analysis.

## 5. Conclusions

The measurement of dielectric properties of food materials is used to quantify the interaction of foods with outer electromagnetic energy during processing. The dielectric properties are affected by the applied frequency, temperature, bulk density, concentration, and the constituents of foods.

Previous studies established the relationship between dielectric properties and their influencing factors using classical regression analysis. The criteria to determine the adequacy of these equations are the coefficient of determination, *R*^2^, and the *p*-value. Modern regression techniques have been developed. The statistical test include tests for data normality, a constant variance, and residual plots.

This study uses sixteen datasets from the literature to establish prediction equations for dielectric properties. The dependent variables are the dielectric constant and the loss factor. The independent variables are the frequency, temperature, and moisture content. The dependent variables and the frequency term are often transformed to establish an appropriate equation for dielectric properties.

This study uses categorical testing to determine the significance of the effect of different conditions on the dielectric properties. These conditions include salted treatment, the position of samples, moisture conditions, and ion concentrations. The results show that this method of categorical testing quantitatively determines the effect. The method can be used for other datasets of dielectric properties to classify the influencing quantitative and qualitative factors. The application of predicted equations of dielectric properties is discussed.

## Figures and Tables

**Table 1:** Published models of dielectric constants for foods.

Study	Food Type	Frequency (MHz)	Temperature (°C)	Moisture Content (%, w.b. or d.b.)	Equations	Statistics Criteria			
						*R* ^2^	*p*-Value	Normal Test	Constant Variance Test
Calay et al. (1995) [22]	Fruit and vegetable	900–3000	0–70	50–90	Equation (1-1)	yes	no	no	no
	Meat	2000–3000	0–70	60–80	Equation (1-2)	yes	no	no	no
	Fish	2450	0–70	>70	Equation (1-3) Equation (1-4)	yes	no	no	no
Guo et al. (2007) [10]	Eggs	10–1800	24	–	Equation (1-5)	yes	no	no	no
Ahmed et al. (2007) [13]	Butter	500–3000	30–80	17–19	Equation (1-6) at fixed temperature	yes	no	no	no
Wang et al. (2007) [17]	Fish (salmon fillets)	27–1800	20–120	74.97–76.14	Equation (1-3)	yes	no	no	no
Dev et al. (2008) [23]	Eggs	20–10,000	0–62		Equation (1-7)	yes	no	no	no
Liu et al. (2009) [14]	Bread	13.56–1800	25–85	34–38.6	Equation (1-8) at fixed temperature and moisture	yes	no	no	no
					Equation (1-9) at fixed temperature and frequency				
					Equation (1-3) at fixed frequency and moisture				
Wang et al. (2009) [9]	Eggs	27–1800	20–120		Equation (1-3) at fixed frequency	yes	no	no	no
Kannan et al. (2013) [24]	Eggs	10–3000	5–56		Egg white: $\varepsilon^{\prime }:$ Equation (1-10)	yes	no	no	no
					Egg white: $\varepsilon^{\prime \prime }$: Equation (1-11)				
					Egg yolk: $\varepsilon^{\prime }$: Equation (1-12)				
					Egg yolk: ε″: Equation (1-13)				
Zhu et al. (2013) [25]	Wheat seeds	1–1000	5–40 °C	11.1–17.1	Equation (1-14)	yes	no	no	no
Boldor et al. (2014) [26]	Peanuts	300–3000	23–50	18–23	Equation (1-4) at fixed frequency	yes	no	no	no
Yu et al. (2015) [27]	Canola seeds	500–3000	30–70	5–11	Equation (1-15)	yes	no	no	no
Zhang et al. (2016) [28]	Peanut kernels	10–4500	25–85	10–30	Equation (1-16)	yes	no	no	no
Boreddy and Subbiah (2016) [8]	Egg white powder	10–3000	20–100	5.5–13.4	Equation (1-17) at fixed frequency	yes	no	no	no
Zhu and Guo (2017) [29]	Potato Starch	20–4500	25–75	15.1–43.1	Equation (1-18) at fixed moisture and temperature	yes	no	no	no
Ling et al. (2018) [30]	Rice bran	300–3000	25–100	10.36–24.69	Equation (1-19)	yes	no	no	no
Zhang et al. (2019) [19]	Pecan Kernels	27–2450	5–65	10–30	Equation (1-20)	yes	no	no	no

Equation (1-1): *y_i_ = a*_0_ *+ a*_1_ *T + a*_2_ *X + a*_3_*f*; Equation (1-2): *y_i_ = b*_0_ *+ b*_1_ *T + b*_2_ *X + b*_12_ *T × X*; Equation (1-3): *y_i_ = c*_0_ *+ c*_1_ *T + c*_2_ *T*^2^; Equation (1-4): *y_i_ = d*_0_ *+ d*_1_ *T + d*_2_ *X*; Equation (1-5): log *y_i_ = e*_0_ *+ e*_1_log *f*; Equation (1-6): *y_i_ = f*_0_ *+ f*_1_*f + f*_2_*f*^2^; Equation (1-7): *y_i_ = g*_0_ *+ g*_1_ *T + g*_2_*f;* Equation (1-8): *y_i_ = h*_0_ *+ h*_1_*/f*; Equation (1-9): *y_i_ = i*_0_ *+ i*_1_ *X*; Equation (1-10): *y_i_ = (c*_0_ *+ c*_1_ *T + c*_2_ *T*^2^*) × exp(j_o_ + j*_1_ *T/f)*; Equation (1-11): *y_i_ = (k*_0_ *+ k*_1_ *T + k*_2_ *T*^2^*)+ (n*_0_ *+ n*_1_ *T + n*_2_ *T*^2^*)/f*; Equation (1-12): *y_i_ = (l*_0_ *+ l*_1_ *T + l*_2_ *T*^2^ *+ l*_3_ *T^3^ + l*_4_ *T*^4^*) × exp*$\left[\left(m_{0}+m_{1}T+m_{2}T^{2}+m_{3}T^{3}+m_{4}T^{4}\right)/f\right]$; Equation (1-13): *y_i_ = (n*_0_ *+ n*_1_ *T + n*_2_ *T*^2^ *+ n*_3_ *T*^3^ *+ n*_4_ *T*^4^*) + (*$o_{0}+o_{1}T+o_{2}T^{2}+o_{3}T^{3}+o_{4}T^{4}$*)/f*; Equation (1-14): *y_i_ =* Multiple linear regression model, variables involved: *(X, T,* ρ*X, T, X ×* ρ*, T ×* ρ*, X*^2^*,* ρ^2^*, T*^2^*, X × T ×* ρ*, X*^2^ *× T, X*^2^ *×* ρ*, X × T*^2^*, X ×* ρ^2^*, T*^2^ *×* ρ*, T*^2^ × ρ^2^*, X^3^, T^3^,* ρ*^3^* at fixed frequency *(*ρ is density); Equation (1-15): *y_i_ =* ρ_0_ *+* ρ_1_*F+*ρ_2_*X +* ρ_3_ *T +* ρ_4_*f ×X +* ρ_5_*f × T +* ρ_6_*X × F+* ρ_7_ *T × X × f;* Equation (1-16): *y_i_* = Multiple linear regression model, variables involved *T, X, T × X, T*^2^ *× X*^2^*, T*^2^ *× X, T × X*^2^*, T^3^, X^3^;* Equation (1-17): *y_i_ = q*_0_ *+ q*_1_ *X + q*_2_ *T + q*_11_ *X*^2^ *+ q*_12_ *T*^2^ *+ q*_12_ *X × T*; Equation (1-18): *y_i_ = r*_0_ *+ r*_1_ log*f*; Equation (1-19): *y_i_* = Multiple linear regression model, variables involved *T, X, X × T, X*^2^*, T*^2^*, X*^2^ *× T, X × T*^2^*, X^3^, T*^3^; Equation (1-20): *y_i_ = t*_0_ *+ t + t*_2_ *T + t*_11_ *X*^2^ *+ t*_22_ *T*^2^ *+ t*_12_ *T × X*; *R*^2^ is the coefficient of determination.

**Table 2:** Published data for dielectric properties in the literature.

Items		Frequency (MHz)						Temperature (°C)								Moisture Content (%)						Literature
Egg	White powder	13.56	27.12	40.68	915	2450		20	40	60	80	100				8	% d.b.					[8]
		27.12	915					20	40	60	80	100				5.5	6.6	8.0	9.8	13.4	% d.b.	
Whites	Liquid	27	40	915	1800			20	40	60	70	80	100	120								[9]
	Precooked																					
Egg	Albumen	10	27	40	100	915	1800	24														[10]
	Yolk	10	27	40	100	915	1800	24														Storage time (0,1,2,3,4,5) weeks
Vegetables	Chickpea flour	27	40	100	915	1800		20	30	40	50	60	70	80	90	7.9	11.4	15.8	20.9	% w.b.		[11]
	Black-eyed peas	27	40	915				20	30	40	50	60				8.8	12.7	16.8	20.9	% w.b.		[12]
Fruits	Apple(GD)	27	40	915	1800			20	30	40	50	60										[20]
	Apple(RD)																					
	Cherry																					
	Grape-fruit																					
	Orange																					
Butter	Unsalted	915	2,450					30	40	50	60	70	80									[13]
	Salted																					
Bread		13.56	27.12	40.68	915	1800		25	40	55	70	85				34.0	34.6	37.1	38.6	% w.b.		[14]
Cheese		270	500	800	1200	1900	3000	5	45	55	65	75	85									[15]
Fish																						
Sturgeon caviar	Unsalted	27	915					20	30	40	50	60	70	80								[16]
	Salted																					
Salmon fillets	Anterior	27	40	915	1800			20	40	60	80	100	120									[17]
	Middle																					
	Tail																					
	Belly																					
Nut	Almond	27	40	915	1800			20	30	40	50	60										[20]
	Walnut	27	40	915	1800			20	30	40	50	60										
	Macadamia nut kernels	27.12	40.68	915	1800			25	40	60	80	100				3	6	12	18	24	% w.b.	[18]
Pecan	Unsalted	27	40	915	2450			5	25	45	65					15	% w.b.					[19]
	Light salted																					
	Medium salted																					
	Heavy salted																					
Insect	Codling moth	27	40	915	1800			20	30	40	50	60										[20]
	Indian-meal moth																					
	Mexican fruit fly																					
	Navel arrange worm																					

**Table 3:** The relationship between the dielectric constant and the influencing factors and statistical criteria established by regression analysis for egg white powder.

Equation (3-1): *ε*′ = 2.928 − (0.0154 × *T*) − (0.000173 × *f*) − (0.274 × *X*) − (0.0000923 × *T*^2^) + (0.0151 × *X*^2^) + (0.0000353 × *T* × *f*) + (0.00558 × *T* × *X*) + (0.00000654 × *f* × *X*) − (0.00000439 × *T* × *f* × *X*)
*R*^2^ = 0.980
Normality Test (Kolmogorov–Smirnov): Passed (*p* = 0.590)
Constant Variance Test (Spearman Rank Correlation): Failed (*p* = 0.041)
Equation (3-2): $\sqrt{\varepsilon {}^{\prime }}$ = 1.432 − (0.000287 × *T*) + (0.0000403 × *f*) − (0.0340 × *X*) − (0.0000334 × *T*^2^) + (0.00279 × *X*^2^) + (0.00000750 × *T* × *f*) + (0.00116 × *T* × *X*) − (0.00000899 × *f* × *X*) − (0.000000906 *T* × *f* × *X*)
*R*^2^ = 0.980
Normality Test: Passed (*p* = 0.398)
Constant Variance Test: Failed (*p* = 0.048)
Equation (3-3): ln *ε*′ = 0.488 + (0.00361 × *T*) + (0.000120 × *f*) + (0.00237 × *X*) − (0.0000460 × *T*^2^) + (0.00175 × *X*^2^) + (0.00000640 × *T* × *f*) + (0.000944 × *T* × *X*) − (0.0000194 × *f* × *X*) − (0.000000743 × *T* × *f* × *X*)
*R*^2^ = 0.976
Normality Test: Passed (*p* = 0.160)
Constant Variance Test: Passed (*p* = 0.497)
Equation (3-4): 1/*ε*′ = 0.678 − (0.00330 × *T*) − (0.0000699 × *f*) − (0.0219 × *X*) + (0.0000203 × *T*^2^) + (0.000155 × *X*^2^) − (0.00000121 × *T* × *f*) − (0.000123 × *T* × *X*) + (0.0000102 × *f* × *X*) + (0.000000119 × *T* × *f* × *X*)
*R*^2^ = 0.990
Normality Test: Passed (*p* = 0.151)
Constant Variance Test: Passed (*p* = 0.467)
Equation (3-5): *ε*′ = 2.954 − (0.0456 × *T*) + (0.0186 × ln *f*) − (0.282 × *X*) − (0.0000926 × *T*^2^) − (0.00778 × ln f^2^) + (0.0150× *X*^2^) + (0.00928 × *T* × ln *f*) + (0.00920 × *T* × *X*) + (0.00273 × ln *f* × *X*) − (0.00113 × *T* × ln *f*) × *X*)
*R*^2^ = 0.980
Normality Test: Passed (*p* = 0.459)
Constant Variance Test: Failed (*p* = 0.003)
Equation (3-6): $\sqrt{\left(\varepsilon {}^{\prime }\right)}$ = 1.390 − (0.00697 × *T*) + (0.0246 × ln *f* − (0.0291 × *X*) − (0.0000335 × *T*^2^) − (0.00231 × ln *f*^2^) + (0.00280 × *X*^2^) + (0.00203 × *T* × ln *f* + (0.00193 × *T* ×* X*) − (0.00181 × ln *f* × *X*) − (0.000236 × T × ln f × *X*)
*R*^2^ = 0.980
Normality Test: Passed (*p* = 0.355)
Constant Variance Test: Failed (*p* = 0.031)
Equation (3-7): ln(*ε*′) = 0.400 − (0.00242 × *T*) + (0.0434 × ln *f* + (0.0140 × *X*) − (0.0000462 × *T*^2^) − (0.00271 × ln *f*^2^) + (0.00180 × *X*^2^) + (0.00181 × *T* × ln *f* + (0.00159 × *T* × *X*) − (0.00422 × ln *f* × *X*) − (0.000197 × *T* × ln *f* × *X*)
*R*^2^ = 0.977
Normality Test: Passed (*p* = 0.370)
Constant Variance Test: Passed (*p* = 0.349)
Equation (3-8): 1/*ε*′ = 0.720 − (0.00196 × *T*) − (0.0199 × ln *f*) − (0.0280 × *X*) + (0.0000204 × *T*^2^) + (0.000905 × ln *f*^2^) + (0.000118 × *X*^2^) − (0.000389 × *T* × ln *f*) − (0.000238 × *T* × *X*) + (0.00227 × ln *f* × *X*) + (0.0000342 × *T* × ln *f* × *X*)
*R*^2^ = 0.964
Normality Test: Passed (*p* = 0.020)
Constant Variance Test: Passed (*p* = 0.574)

**Table 4:** The relationship between the loss factor and the influencing factors and statistical criteria established with regression analysis for egg white powder.

Equation (4-1): ε″ = 1.279 − (0.0230 × *T*) − (0.000605 × *f*) − (0.257 × *X*) − (0.00000381 × *T*^2^) + (0.00862 × *X*^2^) + (0.0000280 × *T* × *f*) + (0.00431 × *T* × *X*) + (0.0000899 × *f* × *X*) − (0.00000363 × *T* × *f* × *X*)
*R*^2^ = 0.965
Normality Test: Passed (*p* = 0.132)
Constant Variance Test: Failed
Equation (4-2): $\sqrt{\varepsilon \prime \prime }$ = 0.124 + (0.00225 × *T*) + (0.0000236 × *f*) − (0.0289 × *X*) − (0.0000489 × *T*^2^*)* + (0.00175 × *X*^2^) + (0.00000957 × *T* × *f*) + (0.00144 × *T* × *X*) + (0.00000852 × *f* × *X*) − (0.00000123 × *T* × *f* × *X*)
*R*^2^ = 0.967
Normality Test: Failed (*p* = 0.006)
Constant Variance Test: Passed (*p* = 0.064)
Equation (4-3): ln(*ε*″) = -5.742 + (0.0599 × *T*) + (0.00112 × *f*) + (0.318 × *X*) − (0.000316 × *T*^2^) − (0.00583 × *X*^2^) + (0.00000753 × *T* × *f*) + (0.000485 × *T* × *X*) − (0.000104 × *f* × *X*) − (0.000000957 × *T* × *f* × *X*)
*R*^2^ = 0.955
Normality Test: Failed (*p* = < 0.001)
Constant Variance Test: Failed (*p* = < 0.001)
Equation (4-4): 1/*ε*″ = 46.309 − (0.612 × *T*) − (0.0100 × *f*) − (4.452 × *X*) + (0.00259 × *T*^2^) + (0.116 × *X*^2^) + (0.0000895 × *T* × *f*) + (0.0238 × *T* × *X*) + (0.00114 × *f* × *X*) − (0.0000109 × *T* × *f* × *X*)
*R*^2^ = 0.712
Normality Test: Failed (*p* = < 0.001)
Constant Variance Test: Failed (*p* = < 0.001)
Equation (4-5): ε″ = 1.667 − (0.0463 × *T*) − (0.336 × *X*) − (0.0688 × ln *f*) − (0.00000442 × *T*^2^) − (0.0105 × ln *f*^2^) + (0.00875 × *X*^2^) + (0.00720 × *T* × ln *f*) + (0.00728 × *T* × *X*) + (0.0234 × ln *f* × *X*) − (0.000923 × *T* × ln *f* × *X*)
*R*^2^ = 0.963
Normality Test: Passed (*p* = 0.01)
Constant Variance Test: Passed (*p* = 0.919)
Equation (4-6): $\sqrt{\varepsilon \prime \prime }$ = 0.115 − (0.00627 × *T*) − (0.0442 × *X*) + (0.0458 × ln *f*) − (0.0000494 × *T*^2^) − (0.00647 × ln *f*^2^) + (0.00197 × *X*^2^) + (0.00259 × *T* × ln *f*) + (0.00247 × *T* × *X*) + (0.00300 × ln *f* × *X*) − (0.000319 × *T* × ln *f* × *X*)
*R*^2^ = 0.964
Normality Test: Failed (*p* = 0.004)
Constant Variance Test: Passed (*p* = 0.098)
Equation (4-7): ln(*ε*″) = −6.414 + (0.0504 × *T*) + (0.369 × *X*) + (0.342 ×ln *f*) − (0.000317 × *T*^2^) − (0.0153 × ln *f*^2^) − (0.00495 × *X*^2^) + (0.00264 × *T* × ln *f*) + (0.00143 × *T* × *X*) − (0.0229 × ln *f* × *X*) − (0.000278 × *T* × ln *f* × *X*)
*R*^2^ = 0.944
Normality Test: Failed (*p* = < 0.001)
Constant Variance Test: Failed (*p* = < 0.001)
Equation (4-8): 1/ε″ = 52.229 − (0.658 × *T*) − (5.242 × *X*) − (2.207 × ln *f*) + (0.00259 × *T*^2^) + (0.0108 × ln *f*^2^) + (0.114 × *X*^2^) + (0.0173 × *T* × ln *f* + (0.0312 × *T* × *X*) + (0.269 × ln *f* × *X*) − (0.00250 × *T* × ln *f* × *X*)
*R*^2^ = 0.952
Normality Test: Failed (*p* = < 0.001)
Constant Variance Test: Failed (*p* = < 0.001)
Equation (4-9): *ε*″ = 1.975 − (0.0471 × *T*) − (0.347 × *X*) − (0.178 × ln *f*) + (0.00932 × *X*^2^) + (0.00723 × *T* × ln *f*) + (0.00729 × *T* × *X*) + (0.0236 × ln *f* × *X*) − (0.000924 × *T* × ln *f* × *X*)
*R*^2^ = 0.962
Normality Test: Passed
Constant Variance Test: Passed (*p* = 0.823)

**Table 5:** The relationship between the dielectric properties and the influencing factors and statistical criteria established by regression analysis for black-eyed peas.

Equation (5-1): ln(*ε*′) = 2.521 − (0.0201 × *T*) − (0.195 × *X*) + (0.0184 × ln *f*) + (0.000242 × *T*^2^) − (0.000327 × ln *f*^2^) + (0.00926 × *X*^2^) + (0.000677 × *T* × ln *f*) + (0.000693 × *T* × *X*) − (0.00662 × ln *f* × *X*) − (0.0000247 × *T* × ln *f* × *X*) − (0.00000124 × (*T* × *X*) ^2^)
*R*^2^ = 0.940
Normality Test: Passed (*p* = 0.310)
Constant Variance Test: Passed (*p* = 0.975)
Equation (5-2): 1/*ε*′ = 0.150 + (0.000411 × *T*) + (0.0268 × *X*) − (0.00544 × ln *f*) − (0.0000337 × *T*^2^) + (0.00136 × ln *f*^2^) − (0.00142 × *X*^2^) + (0.000264 × *T* × ln *f*) + (0.0000185 × *T* × *X*) + (0.000664 × ln *f* × *X*) − (0.0000119 × *T* × ln *f* × *X*) − (0.000000108 × (*T* × *X*) ^2^)
*R*^2^ = 0.928
Normality Test: Passed (*p* = 0.082)
Constant Variance Test: Passed (*p* = 0.897)
Equation (5-3): ln(*ε*″) = 4.531 − (0.0719 × *T*) − (0.375 × *X*) − (1.349 × ln *f*) + (0.000639 × *T*^2^) + (0.0909 × ln *f*^2^) + (0.0131 × *X*^2^) + (0.00674 × *T* × ln *f*) + (0.00315 × *T* × *X*) + (0.0161 × ln *f* × *X*) − (0.0000621 × *T* × ln *f* × *X*) − (0.00000770 × *T* × ln *f*^2^) − (0.000000970 × (*T* × *X*) ^2^)
*R*^2^ = 0.952
Normality Test: Passed (*p* = 0.158)
Constant Variance Test: Passed (*p* = 0.197)

**Table 6:** The relationship between the dielectric properties and the influencing factors and statistical criteria established by regression analysis for macadamia nut kernels.

Equation (6-1): ln(*ε*′) = 1.467 − (0.000659 × *T)* + (0.102 × *X)* − (0.119 × ln *f)* + (0.0000327 × *T*^2^*)* + (0.0170 × ln *f*^2^*)* + (0.00000963 × *X*^2^*)* − (0.000227 × *T* × ln *f)* + (0.000304 × *T* × *X)* − (0.00904 × ln *f* × *X)* - (0.00000325 × *T* × ln *f* × *X)* − (0.000000205 × *T* × ln *f*^2^*)* − (0.0000000679 × (*T* × *X)*^2^)
*R*^2^ = 0.952
Normality Test: Passed (*p* = 0.171)
Constant Variance Test: Failed (*p* = < 0.001)
Equation (6-2): ln(*ε*″) = -1.719 + (0.00660 × *T)* + (0.469 × *X)* − (0.351 × ln *f)* + (0.0000602 × *T*^2^*)* + (0.0627 × ln *f*^2^*)* − (0.00381 × *X*^2^*)* − (0.00102 × *T* × ln *f)* + (0.000778 × *T* × *X)* − (0.0397 × ln *f* × *X*) - (0.000118 × *T* × ln *f* × *X)* + (0.000000144 × (*T* × *X)*^2^)
*R*^2^ = 0.959
Normality Test: Passed (*p* = 0.165)
Constant Variance Test: Passed (*p* = 0.711)

**Table 7:** Results and criteria for the regression analysis for the dielectric properties of five fruits, two nuts, and four insect larvae.

**1. Gold Apple**
Equation (7-1): ln(*ε*′) = 3.691 − (0.000619 × *T*) + (0.269 × ln *f*) − (0.0000114 × *T*^2^) − (0.0256 × ln *f*^2^) − (0.000206 × *T* × ln *f*)
*R*^2^ = 0.900
Normality Test: Passed (*p* = 0.637)
Constant Variance Test: Passed (*p* = 0.235)
Equation (7-2): 1/*ε*′ = 0.0228 + (0.00000228 × *T*) − (0.00404 × ln *f*) + (0.000000220 × *T*^2^) + (0.000382 × ln *f*^2^) + (0.00000354 × *T* × ln *f*)
*R*^2^ = 0.901
Normality Test: Passed (*p* = 0.331)
Constant Variance Test: Passed (*p* = 0.402)
Equation (7-3): ln(*ε*″) = 10.015 + (0.0348 × *T*) − (2.176 × ln *f*) + (0.0000411 × *T*^2^) + (0.154 × ln *f*^2^) − (0.00604 × *T* × ln *f*)
*R*^2^ = 0.997
Normality Test: Passed (*p* = 0.131)
Constant Variance Test: Passed (*p* = 0.011)
**2. Red Apple**
Equation (7-4): ln(*ε*′) = 3.788 − (0.00239 × *T*) + (0.251 × ln *f*) + (0.000000945 × *T*^2^) − (0.0237 × ln *f*^2^) − (0.000144 × *T* × ln *f*)
*R*^2^ = 0.926
Normality Test: Passed (*p* = 0.662)
Constant Variance Test: Passed (*p* = 0.021)
Equation (7-5): $\sqrt{\varepsilon \prime \prime }$ = 25.153 + (0.175 × *T*) − (7.248 × ln *f*) + (0.0000614 × *T*^2^) + (0.588 × ln *f*^2^) − (0.0265 × *T* × ln *f*)
*R*^2^ = 0.996
Normality Test: Passed (*p* = 0.022)
Constant Variance Test: Passed (*p* = 0.131)
**3. Cherry**
Equation (7-6): *ε*′ = 142.581 + (0.120 × *T*) − (21.083 × ln *f*) − (0.00145 × *T*^2^) + (1.624 × ln *f*^2^) − (0.0322 × *T* × ln *f*)
*R*^2^ = 0.936
Normality Test: Passed (*p* = 0.153)
Constant Variance Test: Passed (*p* = 0.343)
Equation (7-7): $\sqrt{\varepsilon {}^{\prime }}$ = 12.262 + (0.00874 × *T*) − (1.118 × ln *f*) − (0.0000921 × *T*^2^) + (0.0858 × ln *f*^2^) − (0.00211 × *T* × ln *f*)
*R*^2^ = 0.941
Normality Test: Passed (*p* = 0.284)
Constant Variance Test: Passed (*p* = 0.738)
Equation (7-8): ln(*ε*′) = 5.083 + (0.00246 × *T*) − (0.236 × ln *f*) − (0.0000234 × *T*^2^) + (0.0181 × ln *f*^2^) − (0.000546 × *T* × ln *f*)
R^2^ = 0.946
Normality Test: Passed (*p* = 0.433)
Constant Variance Test: Passed (*p* = 0.933)
Equation (7-9): 1/*ε*′ = 0.00479 − (0.0000451 × *T*) + (0.00261 × ln *f*) + (0.000000376 × *T*^2^) −
(0.000199 × ln *f*^2^) + (0.00000895 × *T* × ln *f*)
*R*^2^ = 0.954
Normality Test: Passed (*p* = 0.538)
Constant Variance Test: Passed (*p* = 0.518)
Equation (7-10): 1/*ε*″ = 0.0900 + (0.00195 × *T*) − (0.0577 × ln *f*) − (0.0000305 × *T*^2^) + (0.00618 × ln *f*^2^) + (0.000135 × *T* × ln *f*)
*R*^2^ = 0.881
Normality Test: Passed (*p* = 0.019)
Constant Variance Test: Passed (*p* = 0.251)
**4. Grapefruit**
Equation (7-11): $\sqrt{\varepsilon {}^{\prime }}$ = 13.036 + (0.0174 × *T*) − (1.566 × ln *f*) + (0.0000179 × *T*^2^) + (0.136 × ln *f*^2^) −(0.00449 × *T* × ln *f*))
*R*^2^ = 0.823
Normality Test: Passed (*p* = 0.024)
Constant Variance Test: Failed (*p* = < 0.001)
Equation (7-12): ln(*ε*″) = 9.996 + (0.0334 × *T*) − (1.926 × ln *f*) + (0.122 × ln *f*^2^) − (0.00455 × *T* × ln *f*)
*R*^2^ = 0.998
Normality Test: Passed (*p* = 0.495)
Constant Variance Test: Passed (*p* = 0.165)
Equation (7-13): 1/*ε*″ = -0.0353 + (0.000122 × *T*) + (0.00677 × ln *f)* − (0.00000400 × *T*^2^) + (0.00124 × ln *f*^2^) + (0.0000249 × *T* × ln *f*)
*R*^2^ = 0.986
Normality Test: Passed (*p* = 0.324)
Constant Variance Test: Passed (*p* = 0.030)
**5. Orange**
Equation (7-14): *ε*′ = 123.333 − (0.159 × *T*) − (14.544 × ln *f*) − (0.000964 × *T*^2^) + (1.114 × ln *f*^2^) − (0.00126 × *T* × ln *f*)
*R*^2^ = 0.968
Normality Test: Passed (*p* = 0.592)
Constant Variance Test: Passed (*p* = 0.229)
Equation (7-15): $\sqrt{\varepsilon {}^{\prime }}$ = 11.381 − (0.00702 × *T*) − (0.828 × ln *f*) − (0.0000692 × *T*^2^) + (0.0640 × ln *f*^2^) − (0.000325 × *T* × ln *f*)
*R*^2^ = 0.969
Normality Test: Passed (*p* = 0.311)
Constant Variance Test: Passed (*p* = 0.157)
Equation (7-16): ln(*ε*′) = 4.930 − (0.00110 × *T*) − (0.188 × ln *f*) − (0.0000193 × *T*^2^) + (0.0147 × ln*f*^2^) − (0.000135 × *T* × ln *f*)
*R*^2^ = 0.969
Normality Test: Passed (*p* = 0.257)
Constant Variance Test: Passed (*p* = 0.089)
Equation (7-17): 1/*ε*′ = 0.00560 + (0.0000000811 × *T*) + (0.00244 × ln *f*) + (0.000000351 × *T*^2^) − (0.000194 × ln *f*^2^) + (0.00000351 × *T* × ln *f*)
*R*^2^ = 0.970
Normality Test: Passed (*p* = 0.049)Constant Variance Test: Passed (*p* = 0.033)
Equation (7-18): ln(*ε*″) = 8.981 + (0.0369 × *T*) − (1.486 × ln *f*) − (0.0000568 × *T*^2^) + (0.0850 × ln*f*^2^) − (0.00474 × *T* × ln *f*)
*R*^2^ = 0.998
Normality Test: Passed (*p* = 0.075)
Constant Variance Test: Passed (*p* = 0.062)
**6. Almond**
Equation (7-19): $\sqrt{\varepsilon {}^{\prime }}$ = 5.816 − (0.00407 × *T*) − (1.357 × ln *f*) + (0.0000683 × *T*^2^) + (0.112 × ln *f*^2^)
*R*^2^ = 0.381
Normality Test: Passed (*p* = 0.179)
Constant Variance Test: Passed (*p* = 0.133)
Equation (7-20): $\sqrt{\varepsilon \prime \prime }$ = −6.515 − (0.0200 × *T*) + (3.221 × ln *f*) + (0.000132 × *T*^2^) − (0.278 ×ln *f*^2^) +(0.00144 × *T*× ln *f*)
*R*^2^ = 0.879
Normality Test: Passed (*p* = 0.120)
Constant Variance Test: Passed (*p* = 0.605)
Equation (7-21): ln(*ε*″) = −8.572 − (0.0505 × *T*) + (3.826 × ln *f*) + (0.000401 × *T*^2^) − (0.328 × ln *f*^2^) + (0.00268 × *T* × ln *f*)
*R*^2^ = 0.878
Normality Test: Passed (*p* = 0.016)
Constant Variance Test: Passed (*p* = 0.030)
**7. Walnut**
Equation (7-22): *ε*′ = 11.067 − (0.0193 × *T*) − (2.308 × ln *f*) + (0.132 × ln *f*^2^) + (0.00855 × *T* × ln *f*)
*R*^2^ = 0.957
Normality Test: Passed (*p* = 0.046)
Constant Variance Test: Passed (*p* = 0.313)
Equation (7-23): $\sqrt{\varepsilon {}^{\prime }}$ = 3.861 − (0.00278 × *T*) − (0.639 × ln *f*) − (0.0000549 × *T*^2^) + (0.0361 × ln *f*^2^) + (0.00274 × *T* × ln *f*)
*R*^2^ = 0.941
Normality Test: Passed (*p* = 0.028)
Constant Variance Test: Passed (*p* = 0.386)
Equation (7-24): $\sqrt{\varepsilon \prime \prime }$ = −3.502 − (0.00606 × *T*) + (1.810 ×ln *f*) + (0.0000854 × *T*^2^) − (0.149 × ln *f*^2^) − (0.00136 × *T* × ln *f*)
*R*^2^ = 0.936
Normality Test: Passed (*p* = 0.058)
Constant Variance Test: Passed (*p* = 0.084)
Equation (7-25): ln(*ε″)* = −7.867 − (0.0248 × *T*) + (3.187 × ln *f*) + (0.000201 × *T*^2^) − (0.264 × ln *f*^2^) − (0.000704 × *T* × ln *f*)
*R*^2^ = 0.944
Normality Test: Passed (*p* = 0.190) Constant Variance Test: Passed (*p* = 0.559)
**8. Codling Moth**
Equation (7-26): *ε*′ = 127.988 + (0.0726 × *T*) − (22.820 × ln *f*) + (0.00609 × *T*^2^) + (1.757 × ln *f*^2^) − (0.0863 × T × ln *f)*
*R*^2^ = 0.976
Normality Test: Passed (*p* = 0.112)
Constant Variance Test: Passed (*p* = 0.159)
Equation (7-27): ln(*ε*″) = 10.615 + (0.0186 × *T*) − (2.044 × ln *f*) + (0.000174 × *T*^2^) + (0.127 × ln *f*^2^*)* − (0.00339 × *T* × ln *f*)
*R*^2^ = 0.997
Normality Test: Passed (*p* = 0.390)
Constant Variance Test: Passed (*p* = 0.748)
**9. Indian-Meal Moth**
Equation (7-28): $\sqrt{\varepsilon {}^{\prime }}$ = 15.431 + (0.0545 × *T*) − (2.853 × ln *f*) + (0.000248 × *T*^2^) + (0.226 × ln *f*^2^) − (0.0110 × *T* × ln *f*)
*R*^2^ = 0.993
Normality Test: Passed (*p* = 0.137)
Constant Variance Test: Passed (*p* = 0.015)
Equation (7-29): ln(*ε′*) = 5.885 + (0.0117 × *T*) − (0.640 × ln *f*)) + (0.0000574 × *T*^2^) + (0.0478 × ln *f*^2^) − (0.00249 × *T* × ln *f*)
*R*^2^ = 0.996
Normality Test: Passed (*p* = 0.355)
Constant Variance Test: Passed (*p* = 0.939)
Equation (7-30): ln(*ε″)* = 9.086 + (0.00878 × *T*) − (1.360 × ln *f*) + (0.000180 × *T*^2^) + (0.0626 × ln *f*^2^) − (0.00229 × *T* × ln *f*)
*R*^2^ = 0.996
Normality Test: Passed (*p* = 0.296)
Constant Variance Test: Passed (*p* = 0.779)
**10. Mexican Fruit Fly**
Equation (7-31): *ε*′^−1.5^ = -0.00167 − (0.0000389 × *T*) + (0.00125 × ln *f*) − (0.0000862 × ln *f*^2^) + (0.00000678 × *T* × ln *f*)
*R*^2^ = 0.965
Normality Test: Passed (*p* = 0.661)
Constant Variance Test: Passed (*p* = 0.014)
Equation (7-32): *ε*″ = 1222.142 + (9.378 × *T*) − (417.002 ×ln *f*) + (0.0120 × T^2^) + (34.831 × ln *f*^2^) − (1.417 × T × ln *f*)
*R*^2^ = 0.985
Normality Test: Passed (*p* = 0.428)
Constant Variance Test: Passed (*p* = 0.159)
**11. Navel Orange Worm**
Equation (7-33): 1/*ε*′ = -0.00500 − (0.0000968 × *T*) + (0.00703 × ln *f*) − (0.000000520 × *T*^2^) − (0.000456 × ln *f*^2^*)* + (0.0000238 × *T* × ln *f*)
*R*^2^ = 0.997
Normality Test: Passed (*p* = 0.229)
Constant Variance Test: Passed (*p* = 0.126)
Equation (7-34): ln(*ε*″) = 9.662 + (0.0225 × *T*) − (1.520 ×ln *f*)) + (0.0725 × ln *f*^2^) − (0.00213 × *T* × ln *f*)
*R*^2^ = 0.999
Normality Test: Passed (*p* = 0.447)
Constant Variance Test: Passed (*p* = 0.741)

**Table 8:** Study of the effect of the storage time on the dielectric properties of eggs using regression analysis.

**1. Egg Yolk**						
Equation (8-1): ln(*ε*′) = 5.722 − (0.661 × ln *f*) + (0.0498 × ln *f*^2^)						
*R*^2^ = 0.967						
Normality Test: Passed (*p* = 0.451)						
Constant Variance Test: Passed (*p* = 0.270)						
Equation (8-2): ln(*ε*′) = 5.717 − (0.00498 × *St*) − (0.664 × ln *f*)) + (0.00198 × *St*^2^) + (0.0498 × ln *f*^2^) +(0.000975 × *St* × ln *f*)						
*R*^2^ = 0.970						
		**Coefficient**	**Std. Error**	**t**	***p***	
	Constant	5.717	0.106	53.775	<0.001	
	*St*	−0.00498	0.0269	−0.185	0.854	
	ln *f*	−0.664	0.0441	−15.051	<0.001	
	*St* ^2^	0.00198	0.00422	0.468	0.643	
	ln *f*^2^	0.0498	0.00427	11.644	<0.001	
	*St×* ln *f*	0.000975	0.00329	0.297	0.769	
Equation (8-3): ln(*ε″)* = 9.088 − (1.315 × ln *f*)) + (0.0520 × ln *f*^2^)						
*R*^2^ = 0.998						
Equation (8-4): ln(*ε*″) = 9.087 − (0.00768 × *St*) − (1.318 × ln *f)* + (0.00210 × *St*^2^) + (0.0520 × ln *f*^2^) + (0.00113 × *St* × ln *f*)						
*R*^2^ = 0.998						
		**Coefficient**	**Std. Error**	**t**	***p***	
	Constant	9.087	0.109	83.221	<0.001	
	*St*	−0.00768	0.0276	−0.278	0.783	
	ln *f*	−1.318	0.0453	−29.089	<0.001	
	*St* ^2^	0.00210	0.00433	0.484	0.632	
	ln *f*^2^	0.0520	0.00439	11.833	<0.001	
	*St×* ln *f*	0.00113	0.00338	0.336	0.739	
**2. Albumen**						
Equation (8-5): ln(*ε′)* = 6.269 − (0.721 × ln *f*) + (0.0604 × ln *f*^2^)						
*R*^2^ = 0.898						
Equation (8-6): ln(*ε*′) = 6.275 − (0.0216 × *St*) − (0.718 × ln *f*) + (0.00523 × *St*^2^) + (0.0604 × ln *f*^2^) – (0.00126 × *St* × ln *f*)						
*R*^2^ = 0.901						
Equation (8-7): ln(*ε*″) = 10.077 − (1.300 × ln *f*) + (0.0425 × ln *f*^2^)						
*R*^2^ = 0.999						
Equation (8-8): ln(*ε*″) = 10.052 − (0.0107 × *St*) − (1.297 × ln *f)* + (0.000141 × *St*^2^) + (0.0425 × ln *f*^2^) – (0.00108 × St × ln *f)*						
*R*^2^ = 0.999						
		**Coefficient**	**Std. Error**	**t**	***p***	
	Constant	10.052	0.106	94.628	<0.001	
	*St*	0.0107	0.0268	0.398	0.693	
	ln *f*	−1.297	0.0441	−29.432	<0.001	
	*St* ^2^	−0.000141	0.00421	−0.0335	0.973	
	ln *f*^2^	0.0425	0.00427	9.956	<0.001	
	*St×* ln *f*	−0.00108	0.00329	−0.328	0.745	

**Table 9:** Study of the effect of two categories on the dielectric properties of butter and eggs using regression analysis.

**1. Butter (Salted and Unsalted)**						
Equation (9-1): ln(*ε*′) = 3.777 + (1.370 × *z*) + (0.00355 × *T*) + (0.00433 × *T* × *z*) − (0.000160 × *T*^2^) + (0.000151 × *T*^2^ × *z*) − (0.0680 × ln *f*) − (0.349 × ln *f* × *z*)						
*R*^2^ = 0.965						
		**Coefficient**	**Std. Error**	**t**	***p***	
	Constant	3.777	0.206	18.342	<0.001	
	*z*	1.370	0.291	4.706	<0.001	
	*T*	0.00355	0.00488	0.728	0.470	
	*T* × *z*	0.00433	0.00691	0.627	0.533	
	*T* ^2^	−0.000160	0.0000440	−3.647	<0.001	
	*T*^2^ × *z*	0.000151	0.0000622	2.424	0.018	
	ln *f*	−0.0680	0.0223	−3.049	0.003	
	ln *f* × *z*	−0.349	0.0315	−11.065	<0.001	
Equation (9-2): ln *ε*″ = 3.978 + (19.066 × *z*) − (0.0126 × *T*) + (0.0902 × *T* × *z*) − (0.00000579 × *T*^2^) − (0.000241 × *T*^2^ × *z*) − (0.192 × ln *f*) − (2.600 × ln *f*^2^ × *z*)						
*R*^2^ = 0.989						
		**Coefficient**	**Std. Error**	**t**	***p***	
	Constant	3.978	0.851	4.674	<0.001	
	*z*	19.066	1.204	15.839	<0.001	
	*T*	−0.0126	0.0202	−0.623	0.536	
	*T* × *z*	0.0902	0.0285	3.158	0.002	
	*T* ^2^	−0.00000579	0.000182	−0.0318	0.975	
	*T*^2^ × *z*	−0.000241	0.000257	−0.935	0.353	
	ln *f*	−0.192	0.0921	−2.081	0.041	
	ln *f* × *z*	−2.600	0.130	−19.950	<0.001	
**2. Salmon Fish (Salted and Unsalted)**						
Equation (9-3): $\sqrt{\varepsilon {}^{\prime }}$ = 12.759 + (6.579 × *z*) − (0.117 × *T*) + (0.0296 × *T* × *z*) + (0.00105 × *T*^2^) − (0.790 × ln *f*) − (1.128 × ln *f* × *z*)						
*R*^2^ = 0.912						
		**Coefficient**	**Std. Error**	**t**	***p***	
	Constant	12.759	0.837	15.249	<0.001	
	*z*	6.579	0.800	8.221	<0.001	
	*T*	−0.117	0.0302	−3.887	<0.001	
	*T* × *z*	0.0296	0.0102	2.911	0.005	
	*T* ^2^	0.00105	0.000293	3.576	<0.001	
	ln *f*	−0.790	0.0816	−9.680	<0.001	
	ln *f* × *z*	−1.128	0.115	−9.769	<0.001	
Equation (9-4): ln ε″ = 9.649 + (1.244 × z) − (0.0301 × T) + (0.00760 × T × z) + (0.000343 × *T*^2^) − (0.935 × ln *f*) − (0.0831 × ln *f* × *z*)						
*R*^2^ = 0.973						
		**Coefficient**	**Std. Error**	**t**	***p***	
	Constant	9.649	0.288	33.473	<0.001	
	*z*	1.244	0.276	4.513	<0.001	
	*T*	−0.0301	0.0104	−2.896	0.005	
	*T* × *z*	0.00760	0.00350	2.170	0.033	
	*T* ^2^	0.000343	0.000101	3.396	0.001	
	ln *f*	−0.935	0.0281	−33.245	<0.001	
	ln *f* × *z*	−0.0831	0.0398	−2.090	0.040	

**Table 10:** Study of the effect of three categories on the dielectric properties of cheese using regression analysis.

Moisture Content of Cheese: High, Medium and Low						
Equation (10-1): $\sqrt{\varepsilon {}^{\prime }}$ = 8.985 + (0.715 × *z*1) + (0.168 × *z*2) − (0.0322 × *T*) + (0.000270 × *T*^2^) − (0.494 × ln *f*) + (0.0231 × *T* × *z*1) − (0.000224 × *T*^2^ × *z*3) + (0.0183 × *T* × *z*2) − (0.000142 × *T*^2^ × *z*2)						
*R*^2^ = 0.932						
		**Coefficient**	**Std. Error**	**t**	***p***	
	Constant	8.985	0.173	52.025	<0.001	
	*z*1	0.715	0.122	5.875	<0.001	
	*z*2	0.168	0.123	1.362	0.176	
	*T*	−0.0322	0.00461	−6.978	<0.001	
	*T* ^2^	0.000270	0.0000495	5.446	<0.001	
	ln *f*	−0.494	0.0218	−22.714	<0.001	
	*T* × *z*1	0.0231	0.00625	3.693	<0.001	
	*T*^2^ × *z*1	−0.000224	0.0000674	−3.326	0.001	
	*T* × *z*2	0.0183	0.00652	2.807	0.006	
	*T*^2^ × *z*2	−0.000142	0.0000701	−2.023	0.046	
Equation (10-2): $\sqrt{\varepsilon \prime \prime }$ = 22.116 + (2.584 × *z*1) − (10.126 × *z*2) + (0.0445 × *T*) − (0.975 × *T*^2^) − (2.178 × ln *f*) − (0.0293 × *T* × *z*1) + (0.864 × *T*^2^ × *z*1) − (0.437 × ln *f* × *z*1) − (0.0335 × *T* × *z*2) + (0.928 × T^2^ × *z*2) + (1.143 × ln *f* × *z*2)						
*R*^2^ = 0.936						
		**Coefficient**	**Std. Error**	**t**	***p***	
	Constant	22.116	0.953	23.203	<0.001	
	*z*1	2.584	1.351	1.913	0.059	
	*z*2	−10.126	1.366	−7.412	<0.001	
	*T*	0.0445	0.00910	4.886	<0.001	
	*T* ^2^	−0.975	0.252	−3.863	<0.001	
	ln *f*	−2.178	0.113	−19.273	<0.001	
	*T* × *z*1	−0.0293	0.0128	−2.282	0.025	
	*T*^2^ × *z*1	0.864	0.357	2.420	0.018	
	ln *f* × *z*1	−0.437	0.160	−2.733	0.008	
	*T* × *z*2	−0.0335	0.0129	−2.591	0.011	
	*T*^2^ × *z*2	0.928	0.360	2.579	0.012	
	ln *f* × *z*2	1.143	0.163	7.009	<0.001	

**Table 11:** Study of the effect of four categories on the dielectric properties of salmon fillets using regression analysis.

Position: Anterior, Middle, Tail, Belly						
Equation (11-1): ln(*ε*′) = 5.719 − (0.0802 × *z*1) + (0.0703 × *z*2) + (0.0585 × *z*3) + (0.00917 × *T*) − (0.487 × ln *f*) + (0.0352 × ln *f*^2^) − (0.00160 × *T* × ln *f*) + (0.000522 × *T* × *z*1) + (0.000684 × *T* × *z*3) + (0.0140 × ln *f* × *z*1) − (0.0160 × ln *f* × *z*3)						
*R*^2^ = 0.973						
		**Coefficient**	**Std. Error**	**t**	***p***	
	Constant	5.719	0.0693	82.472	<0.001	
	*z*1	−0.0802	0.0352	−2.277	0.023	
	*z*2	0.0703	0.0101	6.936	<0.001	
	*z*3	0.0585	0.0352	1.660	0.098	
	*T*	0.00917	0.000372	24.652	<0.001	
	ln *f*	−0.487	0.0265	−18.371	<0.001	
	ln *f*^2^	0.0352	0.00245	14.334	<0.001	
	*T* × ln *f*	−0.00160	0.0000623	−25.612	<0.001	
	*T* × *z*1	0.000522	0.000257	2.029	0.043	
	*T* × *z*3	0.000684	0.000257	2.659	0.008	
	ln *f* × *z*1	0.0140	0.00522	2.685	0.008	
	ln *f* × *z*3	−0.0160	0.00522	−3.066	0.002	
Equation (11-2): ln(*ε*″) = 6.368 − (0.341 × *z*1) − (0.0207 × *z*2) − (0.389 × *z*3) − (0.00129 × *T*) − (0.844 × ln *f*) + (0.179 × ln *f*^2^) − (0.000154 × *T* × ln *f*) + (0.105 × ln *f* × *z*1) + (0.0223 × ln *f* × *z*2) + (0.0743 × ln *f* × *z*3)						
*R*^2^ = 0.766						
		**Coefficient**	**Std. Error**	**t**	***p***	
	Constant	6.368	1.870	3.405	<0.001	
	*z*1	−0.341	0.749	−0.455	0.650	
	*z*2	−0.0207	0.749	−0.0276	0.978	
	*z*3	−0.389	0.749	−0.520	0.603	
	*T*	−0.00129	0.000399	−3.220	0.001	
	ln *f*	−0.844	0.949	−0.890	0.374	
	ln *f*^2^	0.179	0.121	1.485	0.138	
	*T* × ln *f*	−0.000154	0.0000965	−1.593	0.112	
	ln *f* × *z*1	0.105	0.180	0.580	0.562	
	ln *f* × *z*2	0.0223	0.181	0.123	0.902	
	ln *f* × *z*3	0.0743	0.1801	0.411	0.682	

**Table 12:** Study of the effect of four categories on the dielectric properties of pecan nut using regression analysis.

Treatments: No Salted, Light Salted, Medium Salted and Heavy Salted						
Equation (12-1): ln(ε′) = 2.150 − (0.533 × *z*1) − (14.653 × *z*2) − (8.721 × *z*3) + (0.000199 × *T*) + (0.138 × ln *f*) − (0.0287 × ln *f*^2^) + (0.000000720 × *T*^2^) − (0.000602 × *T* × ln *f*) + (0.0257 × *T* × *z*1) − (0.000158 × *T* × *z3)* + (6.999 × ln *f*× *z*2) + (4.786 × ln *f* × *z*3) − (0.661 × ln *f*^2^ × *z*2) − (0.468 × ln *f*^2^ × *z*3)						
*R*^2^ = 0.625						
		**Coefficient**	**Std. Error**	**t**	***p***	
	Constant	2.150	2.034	1.057	0.291	
	*z*1	−0.533	0.321	−1.661	0.098	
	*z*2	−14.653	2.708	−5.411	<0.001	
	*z*3	−8.721	2.495	−3.495	<0.001	
	*T*	0.000199	0.000857	0.232	0.817	
	ln *f*	0.138	0.840	0.164	0.870	
	ln *f*^2^	−0.0287	0.0765	−0.375	0.708	
	*T* ^2^	0.00000072	0.000000235	3.060	0.002	
	*T* × ln *f*	−0.000602	0.000181	−3.327	<0.001	
	*T* × *z*1	0.0257	0.00681	3.781	<0.001	
	*T* × *z*3	−0.000158	0.000220	−0.718	0.473	
	ln *f* × *z*2	6.999	1.094	6.397	<0.001	
	ln *f* × *z*3	4.786	1.022	4.684	<0.001	
	ln *f*^2^ × *z*2	−0.661	0.0991	−6.667	<0.001	
	ln *f*^2^ × *z*3	−0.468	0.0932	−5.022	<0.001	
Equation (12-2): ln(*ε*″) = -7.341 + (6.370 × *z1)* − (4.537 × *z*2) + (2.928 × *z*3) − (0.00236 × *T*) + (4.163 × ln *f*) − (0.448 × ln *f*^2^) + (0.000000707 × *T*^2^) + (0.0000175 × *T* × ln *f*) + (0.0321 × *T* × *z*1) − (3.082 × ln *f* × *z*1) + (2.987 × ln *f* × *z*2) + (0.302 ×ln *f*^2^ × *z*1) − (0.281 × ln *f*^2^ × *z*2)						
*R*^2^ = 0.694						
		**Coefficient**	**Std. Error**	**t**	***p***	
	Constant	−7.341	1.276	−5.755	<0.001	
	*z*1	6.370	3.322	1.918	0.056	
	*z*2	−4.537	2.110	−2.150	0.032	
	*z*3	2.928	0.204	14.335	<0.001	
	*T*	−0.00236	0.000453	−5.215	<0.001	
	ln *f*	4.163	0.532	7.827	<0.001	
	ln *f*^2^	−0.448	0.0503	−8.903	<0.001	
	*T* ^2^	0.000000707	0.000000251	2.81	0.005	
	*T* × ln *f*	0.0000175	0.00000289	6.059	<0.001	
	*T* × *z*1	0.0321	0.00767	4.182	<0.001	
	ln *f* × *z*1	−3.082	1.371	−2.249	0.025	
	ln *f* × *z*2	2.987	0.836	3.575	<0.001	
	ln *f*^2^ × *z*1	0.302	0.125	2.413	0.016	
	ln *f*^2^ × *z*2	−0.281	0.0756	−3.723	<0.001	

## Supplementary Materials

The following are available online at https://www.mdpi.com/2304-8158/9/10/1472/s1, Table S1: title, Video S1: The relationship between the dielectric constant and the influencing factors and statistical criteria established by regression analysis for chickpea flour; Table S2: The relationship between the loss factor and the influencing factors and statistical criteria established by regression analysis for chickpea flour; Table S3. The relationship between the dielectric constant and the influencing factors and statistical criteria established by regression analysis for white bread; Table S4. The relationship between the loss factor and the influencing factors and statistical criteria established by regression analysis for white bread; Table S5. The relationship between the dielectric properties and the influencing factors and statistical criteria established by regression analysis for two types of egg white.
