# Supplementary material for: Use of Modern Regression Analysis in the Dielectric Properties of Foods
Source: Foods. 2020 Oct 15;9(10):1472. doi: 10.3390/foods9101472 (PMC7602722; doi:10.3390/foods9101472)
Supplement: Supplementary file 1 [file foods-09-01472-s001.pdf]

Table S1. The relationship between the dielectric constant and the influencing factors and statistical criteria established by regression analysis for chickpea flour.

Equation S1-1: $\varepsilon' = 39.068 - (1.091 \times T) - (0.0120 \times f) - (3.043 \times X) + (0.00592 \times T^2) + (0.00000342 \times f^2) + (0.0442 \times X^2) + (0.000192 \times T \times f) + (0.0589 \times T \times X) + (0.000754 \times f \times X) - (0.0000242 \times T \times f \times X)$
$R^2 = 0.898$
Normality Test: Failed ($p = < 0.001$)
Constant Variance Test: Failed ($p = < 0.001$)
Equation S1-2: $\sqrt{\varepsilon'} = 4.364 - (0.0968 \times T) - (0.000945 \times f) - (0.228 \times X) + (0.000609 \times T^{2)} + (0.000000381 \times f^{2)} + (0.00340 \times X^{2)} + (0.0000101 \times T \times f) + (0.00564 \times T \times X) + (0.0000411 \times f \times X) - (0.00000168 \times T \times f \times X)$
$R^2 = 0.948$
Normality Test: Failed ($p = < 0.001$)
Constant Variance Test: Failed ($p = < 0.001$)
Equation S1-3: $\ln(\varepsilon') = 1.437 - (0.0298 \times T) - (0.000352 \times f) - (0.0379 \times X) + (0.000267 \times T^{2)} + (0.000000204 \times f^{2)} + (0.000799 \times X^{2)} - (0.000000503 \times T \times f) + (0.00213 \times T \times X) - (0.00000149 \times f \times X) - (0.000000282 \times T \times f \times X)$
$R^2 = 0.960$
Normality Test: Passed ($p = 0.017$)
Constant Variance Test: Failed) ($p = <0.001$)
Equation S1-4: $1/\varepsilon' = 0.560 - (0.00263 \times T) + (0.0000684 \times f) - (0.0161 \times X) - (0.0000110 \times T^{2)} - (0.0000000260 \times f^{2)} + (0.000117 \times X^{2)} + (0.000000459 \times T \times f) + (0.00000309 \times T \times X) + (0.00000239 \times f \times X) - (0.0000000544 \times T \times f \times X)$
$R^2 = 0.941$
Normality Test: Passed ($p = 0.039$)
Constant Variance Test: Passed ($p = 0.091$)
Equation S1-5: $\varepsilon' = 59.627 - (1.443 \times T) - (7.004 \times \ln f) - (4.453 \times X) + (0.00581 \times T^{2)} + (0.379 \times \ln f^{2)} + (0.0442 \times X^{2)} + (0.0919 \times T \times \ln f) + (0.104 \times T \times X) + (0.358 \times \ln f \times X) - (0.0115 \times T \times \ln f \times X)$
$R^2 = 0.898$
Normality Test: Failed ($p = < 0.001$)
Constant Variance Test (Spearman Rank Correlation): Failed ($p = < 0.001$)
Equation S1-6: $\sqrt{\varepsilon'} = 5.492 - (0.115 \times T) - (0.422 \times \ln f) - (0.304 \times X) + (0.000599 \times T^{2)} + (0.0268 \times \ln f^{2)} + (0.00340 \times X^{2)} + (0.00479 \times T \times \ln f) + (0.00871 \times T \times X) + (0.0193 \times \ln f \times X) - (0.000782 \times T \times \ln f \times X)$
$R^2 = 0.964$
Normality Test: Failed ($p = < 0.001$)
Constant Variance Test: Failed ($p = < 0.001$)
Equation S1-7: $\ln(\varepsilon') = 1.527 - (0.0286 \times T) - (0.0594 \times \ln f) - (0.0354 \times X) + (0.000263 \times T^{2)} + (0.00581 \times \ln f^{2)} + (0.000799 \times X^{2)} - (0.000206 \times T \times \ln f) + (0.00263 \times T \times X) - (0.000603 \times \ln f \times X) - (0.000130 \times T \times \ln f \times X)$
$R^2 = 0.965$
Normality Test: Failed ($p = 0.002$)
Constant Variance Test: Failed($p = < 0.001$)
Equation S1-8: $1/\varepsilon' = 1.267 - (0.00599 \times T) + (0.0269 \times \ln f) - (0.0356 \times X) - (0.0000162 \times T^{2)} + (0.00266 \times \ln f^{2)} + (0.000245 \times X^{2)} - (0.0000889 \times T \times \ln f + (0.000110 \times T \times X) + (0.000312 \times \ln f \times X) - (0.0000217 \times T \times \ln f \times X)$
$R^2 = 0.951$
Normality Test: Failed ($p = 0.002$)
Constant Variance Test: Passed ($p = 0.799$)

Table S2. The relationship between the loss factor and the influencing factors and statistical criteria established by regression analysis for chickpea flour.

Equation S2-1: $\varepsilon'' = 130.867 - (3.114 \times \mathrm{T}) - (0.0530 \times f) - (11.887 \times X) + (0.0138 \times T^{2)} + (0.00000984 \times f^{2} + (0.208 \times X^{2)} + (0.00101 \times T \times f) + (0.167 \times T \times X) + (0.00371 \times f \times X) - (0.000105 \times T \times f \times X)$
$R^2 = 0.688$
Normality Test: Failed ($p = < 0.001$)
Constant Variance Test: Failed ($p = < 0.001$)
Equation S2-2: $\sqrt{\varepsilon''} = 8.247 - (0.217 \times T) - (0.00321 \times f) - (0.726 \times X) + (0.00107 \times T^{2)} + (0.000000841 \times f^{2)} + (0.0127 \times X^{2)} + (0.0000591 \times T \times f) + (0.0126 \times T \times X) + (0.000208 \times f \times X) - (0.00000676 \times T \times f \times X)$
$R^2 = 0.882$
Normality Test: Failed ($p = < 0.001$)
Constant Variance Test: Failed ($p = < 0.001$)
Equation S2-3: $\ln(\varepsilon'') = -2.028 - (0.0476 \times T) - (0.000381 \times f) - (0.00853 \times X) + (0.000523 \times T^{2)} + (0.000000422 \times f^{2)} + (0.00226 \times X^{2)} + (0.00000614 \times T \times f) + (0.00379 \times T \times X) + (0.00000475 \times f \times X) - (0.00000149 \times T \times f \times X)$
$R^2 = 0.941$
Normality Test: Failed ($p = 0.006$)
Constant Variance Test: Failed ($p = < 0.001$)
Equation S2-4: $1/\varepsilon'' = 15.518 - (0.132 \times T) - (0.00149 \times f) - (0.852 \times X) - (0.000139 \times T^{2)} - (0.00000000181 \times f^{2)} + (0.00794 \times X^{2)} + (0.0000152 \times T \times f) + (0.00601 \times T \times X) + (0.0000897 \times f \times X) - (0.000000878 \times T \times f \times X)$
$R^2 = 0.754$
Normality Test: Failed ($p = < 0.001$)
Constant Variance Test: Failed ($p = < 0.001$)
Equation S2-5: $\varepsilon'' = 242.855 - (5.054 \times T) - (19.071 \times X) - (35.980 \times \ln f) + (0.0134 \times T^{2)} + (1.707 \times \ln f^{2)} + (0.208 \times X^{2)} + (0.497 \times T \times \ln f) + (0.371 \times T \times X) + (1.809 \times \ln f \times X) - (0.0511 \times T \times \ln f \times X)$
$R^2 = 0.774$
Normality Test: Failed ($p = < 0.001$)
Constant Variance Test: Failed ($p = < 0.001$)
Equation S2-6: $\sqrt{\varepsilon''} = 13.821 - (0.321 \times T) - (1.104 \times X) - (1.897 \times \ln f) + (0.00104 \times T^{2)} + (0.101 \times \ln f^{2)} + (0.0127 \times X^{2)} + (0.0274 \times T \times \ln f) + (0.0250 \times T \times X) + (0.0967 \times \ln f \times X) - (0.00315 \times T \times \ln f \times X)$
$R^2 = 0.933$
Normality Test: Failed ($p = < 0.001$)
Constant Variance Test: Failed ($p = < 0.001$)
Equation S2–7: $\ln(\varepsilon'') = 3.935 - (0.268 \times T) - (0.339 \times X) - (0.595 \times \ln f) + (0.00300 \times T^{2)} + (0.0584 \times \ln f^{2)} + (0.00903 \times X^{2)} + (0.00888 \times T \times \ln f) + (0.0122 \times T \times X) - (0.000623 \times T \times \ln f \times X) - (0.0000115 \times T^{3)} - (0.00000589 \times (T \times f^{2)} - (0.00000191 \times (T \times X)^{2})$
$R^2 = 0.962$
Normality Test: Passed ($p = < 0.001$)
Constant Variance Test: Passed ($p = 0.833$)
Equation S2-8: $1/\varepsilon'' = 18.385 - (0.163 \times T) - (1.007 \times X) - (0.759 \times \ln f) - (0.000139 \times T^{2)} + (0.00702 \times \ln f^{2)} + (0.00794 \times X^{2)} + (0.00760 \times T \times \ln f) + (0.00770 \times T \times X) + (0.0399 \times \ln f \times X) - (0.000424 \times T \times \ln f \times X)$
$R^2 = 0.755$
Normality Test: Failed ($p = < 0.001$)
Constant Variance Test: Failed ($p = < 0.001$)

Table S3. The relationship between the dielectric constant and the influencing factors and statistical criteria established by regression analysis for white bread.

Equation S3-1: $\varepsilon' = -4.225 + (0.0695 \times T) - (0.000370 \times f) + (0.184 \times X) + (0.0000754 \times T^{2}) + (0.000000676 \times f^{2}) - (0.00000878 \times T \times f) - (0.00167 \times T \times X) - (0.0000281 \times f \times X)$
$R^2 = 0.844$
Normality Test: Failed $p = <0.001$)
Constant Variance Test: Failed ($p = < 0.001$)
Equation S3-2: $\sqrt{\varepsilon'} = -0.508 + (0.0226 \times T) - (0.000217 \times f) + (0.0569 \times X) + (0.0000177 \times T^{2}) + (0.000000205 \times f^{2}) - (0.00000229 \times T \times f) - (0.000543 \times T \times X) - (0.00000630 \times f \times X)$
$R^2 = 0.870$
Normality Test: Failed ($p = < 0.001$)
Constant Variance Test: Failed ($p = < 0.001$)
Equation S3–3: $\ln(\varepsilon') = -1.699 + (0.0295 \times T) - (0.000404 \times f) + (0.0711 \times X) + (0.0000160 \times T^{2}) + (0.000000252 \times f^{2}) - (0.00000233 \times T \times f) - (0.000709 \times T \times X) - (0.00000473 \times f \times X)$
$R^2 = 0.892$
Normality Test: Failed ($p = < 0.001$)
Constant Variance Test: Failed ($p = < 0.001$)
Equation S3-4: $1/\varepsilon' = 1.448 - (0.0127 \times T) + (0.000273 \times f) - (0.0284 \times X) - (0.00000243 \times T^{2}) - (0.0000000965 \times f^{2}) + (0.000000524 \times T \times f) + (0.000307 \times T \times X) - (0.000000780 \times f \times X)$
$R^2 = 0.924$
Normality Test: Passed ($p = 0.139$)
Constant Variance Test: Failed ($p = < 0.001$)
Equation S3-5: $\varepsilon' = -3.421 + (0.0815 \times T) + (0.222 \times X) - (0.590 \times \ln f) + (0.0000754 \times T^{2}) + (0.0882 \times \ln f^{2}) - (0.00352 \times T \times \ln f) - (0.00167 \times T \times X) - (0.0113 \times \ln f \times X)$
$R^2 = 0.958$
Normality Test: Failed ($p = 0.002$)
Constant Variance Test: Failed ($p = < 0.001$)
Equation S3-6: $\sqrt{\varepsilon'} = -0.196 + (0.0257 \times T) + (0.0653 \times X) - (0.185 \times \ln f) + (0.0000177 \times T^{2}) + (0.0231 \times \ln f^{2}) - (0.000904 \times T \times \ln f) - (0.000543 \times T \times X) - (0.00249 \times \ln f \times X)$
$R^2 = 0.965$
Normality Test: Passed ($p = 0.014$)
Constant Variance Test: Failed ($p = < 0.001$)
Equation S3-7: $\ln(\varepsilon') = -1.217 + (0.0325 \times T) + (0.0772 \times X) - (0.240 \times \ln f) + (0.0000160 \times T^{2}) + (0.0241 \times \ln f^{2}) - (0.000905 \times T \times \ln f) - (0.000709 \times T \times X) - (0.00181 \times \ln f \times X)$
$R^2 = 0.970$
Normality Test: Passed ($p = 0.028$)
Constant Variance Test: Failed ($p = < 0.001$)
Equation S3-8: $1/\varepsilon' = 1.169 - (0.0133 \times T) - (0.0272 \times X) + (0.109 \times \ln f) - (0.00000243 \times T^{2}) - (0.00626 \times \ln f^{2}) + (0.000194 \times T \times \ln f) + (0.000307 \times T \times X) - (0.000353 \times \ln f \times X)$
$R^2 = 0.974$
Normality Test: Passed ($p = 0.095$)
Constant Variance Test: Passed ($p = 0.560$)
Best equation
Equation S3-9: $1/\varepsilon' = 1.236 - (0.0136 \times T) - (0.0289 \times X) + (0.0959 \times \ln f) - (0.00626 \times \ln f^2) + (0.000194 \times T \times \ln f) + (0.000307 \times T \times X)$
$R^2 = 0.974$
Normality Test: Passed ($p = 0.356$)
Constant Variance Test: Passed ($p = 0.863$)

Table S4. The relationship between the loss factor and the influencing factors and statistical criteria established by regression analysis for white bread.

Equation S4-1: $\varepsilon'' = -37.684 + (0.217 \times T) + (0.0107 \times f) + (1.121 \times X) + (0.00164 \times T^{2}) + (0.00000571 \times f^{2}) - (0.000131 \times T \times f) - (0.00542 \times T \times X) - (0.000519 \times f \times X)$
$R^2 = 0.691$
Normality Test: Failed $p = < 0.001$)
Constant Variance Test: Failed ($p = < 0.001$)
Equation S4-2: $\sqrt{\varepsilon''} = -8.171 + (0.0906 \times T) + (0.000577 \times f) + (0.269 \times X) + (0.000169 \times T^{2}) + (0.00000128 \times f^{2}) - (0.0000197 \times T \times f) - (0.00210 \times T \times X) - (0.0000828 \times f \times X)$
$R^2 = 0.845$
Normality Test: Failed ($p = < 0.001$)
Constant Variance Test: Failed ($p = < 0.001$)
Equation S4-3: $\ln(\varepsilon'') = -9.622 + (0.124 \times T) - (0.00211 \times f) + (0.290 \times X) + (0.0000507 \times T^{2}) + (0.00000139 \times f^{2}) - (0.0000102 \times T \times f) - (0.00291 \times T \times X) - (0.0000345 \times f \times X)$
$R^2 = 0.841$
Normality Test: Failed ($p = < 0.001$)
Constant Variance Test: Failed ($p = < 0.001$)
Equation S4-4: $1/\varepsilon'' = 6.225 - (0.0903 \times T) + (0.00496 \times f) - (0.159 \times X) + (0.0000207 \times T^{2}) - (0.000000683 \times f^{2}) - (0.00000671 \times T \times f) + (0.00229 \times T \times X) - (0.0000694 \times f \times X)$
$R^2 = 0.960$
Normality Test: Failed ($p = < 0.001$)
Constant Variance Test: Failed ($p = < 0.001$)
Equation S4-5: $\varepsilon'' = -38.906 + (0.412 \times T) + (1.901 \times X) - (5.157 \times \ln f) + (0.00164 \times T^{2}) + (1.362 \times \ln f^{2}) - (0.0560 \times T \times \ln f) - (0.00542 \times T \times X) - (0.224 \times \ln f \times X)$
$R^2 = 0.920$
Normality Test: Passed ($p = 0.015$)
Constant Variance Test: Failed ($p = < 0.001$)
Equation S4-6: $\sqrt{\varepsilon''} = -8.256 + (0.118 \times T) + (0.386 \times X) - (0.703 \times \ln f) + (0.000169 \times T^{2}) + (0.180 \times \ln f^{2}) - (0.00796 \times T \times \ln f) - (0.00210 \times T \times X) - (0.0342 \times \ln f \times X)$
$R^2 = 0.980$
Normality Test: Passed ($p = 0.429$)
Constant Variance Test: Failed ($p = < 0.001$)
Equation S4-7: $\ln(\varepsilon'') = -8.366 + (0.136 \times T) + (0.336 \times X) - (0.716 \times \ln f) + (0.0000507 \times T^{2}) + (0.0793 \times \ln f^{2}) - (0.00369 \times T \times \ln f) - (0.00291 \times T \times X) - (0.0137 \times \ln f \times X)$
$R^2 = 0.993$
Normality Test: Passed ($p = 0.017$)
Constant Variance Test: Passed ($p = 0.985$)
Equation S4-8: $1/\varepsilon'' = 2.285 - (0.0802 \times T) - (0.0725 \times X) + (1.093 \times \ln f) + (0.0000207 \times T^{2}) + (0.0344 \times \ln f^{2}) - (0.00291 \times T \times \ln f) + (0.00229 \times T \times X) - (0.0262 \times \ln f \times X)$
$R^2 = 0.968$
Normality Test: Failed ($p = < 0.001$)
Constant Variance Test: Failed ($p = < 0.001$)

Table S5. The relationship between the dielectric properties and the influencing factors and statistical criteria established by regression analysis for two types of egg white.

**(I) Liquid**
Equation S5-1: $\varepsilon' = 191.437 + (0.160 \times T) - (47.142 \times \ln f) + (0.00505 \times T^2) + (4.390 \times \ln f^2) - (0.134 \times T \times \ln f)$
$R^2 = 0.904$
Normality Test: Passed ($p = 0.420$)
Constant Variance Test: Failed ($p = < 0.001$)
Equation S5-2: $\sqrt{\varepsilon'} = 15.047 + (0.00612 \times T) - (2.528 \times \ln f) + (0.000279 \times T^2) + (0.231 \times \ln f^2) - (0.00716 \times T \times \ln f)$
$R^2 = 0.920$
Normality Test: Passed ($p = 0.787$)
Constant Variance Test: Failed ($p = 0.021$)
Equation S5-3: $\ln(\varepsilon') = 5.736 + (0.000700 \times T) - (0.548 \times \ln f) + (0.0000632 \times T^2) + (0.0490 \times \ln f^2) - (0.00155 \times T \times \ln f)$
$R^2 = 0.928$
Normality Test: Passed ($p = 0.579$)
Constant Variance Test: Passed ($p = 0.304$)
Equation S5-4: $1/\varepsilon' = -0.00480 + (0.0000107 \times T) + (0.00662 \times \ln f) - (0.000000872 \times T^2) - (0.000565 \times \ln f^2) + (0.0000193 \times T \times \ln f)$
$R^2 = 0.927$
Normality Test: Passed ($p = 0.520$)
Constant Variance Test: Passed ($p = 0.140$)
Equation S5-5: $\varepsilon'' = 2238.665 + (12.754 \times T) - (861.803 \times \ln f) + (0.0443 \times T^2) + (79.932 \times \ln f^2) - (2.615 \times T \times \ln f)$
$R^2 = 0.953$
Normality Test: Passed ($p = 0.148$)
Constant Variance Test: Failed ($p = < 0.001$)
Equation S5-6: $\sqrt{\varepsilon''} = 61.699 + (0.243 \times T) - (18.220 \times \ln f) + (0.000604 \times T^2) + (1.445 \times \ln f^2) - (0.0423 \times T \times \ln f)$
$R^2 = 0.990$
Normality Test: Passed ($p = 0.820$)
Constant Variance Test: Failed ($p = < 0.001$)
Equation S5-7: $\ln(\varepsilon'') = 9.692 + (0.0115 \times T) - (1.322 \times \ln f) + (0.0000486 \times T^2) + (0.0478 \times \ln f^2) - (0.00124 \times T \times \ln f)$
$R^2 = 0.997$
Normality Test: Passed ($p = 0.777$)
Constant Variance Test: Passed ($p = 0.115$)
Equation S5-8: $1/\varepsilon'' = 0.0547 + (0.000424 \times T) - (0.0325 \times \ln f) - (0.000000779 \times T^2) + (0.00483 \times \ln f^2) - (0.0000979 \times T \times \ln f)$
$R^2 = 0.970$
Normality Test: Failed ($p = < 0.001$)
Constant Variance Test: Failed ($p = < 0.001$)
**(II) Precooked**
Equation S5-9: $\varepsilon' = 181.838 + (0.278 \times T) - (39.590 \times \ln f) + (0.00242 \times T^2) + (3.427 \times \ln f^2) - (0.107 \times T \times \ln f)$
$R^2 = 0.959$
Normality Test: Failed ($p = < 0.001$)
Constant Variance Test: Failed ($p = 0.018$)
Equation S5-10: $\sqrt{\varepsilon'} = 14.398 + (0.0155 \times T) - (2.094 \times \ln f) + (0.000127 \times T^2) + (0.178 \times \ln f^2) - (0.00606 \times T \times \ln f)$
$R^2 = 0.969$
Normality Test Failed: ($p = 0.011$)
Constant Variance Test: Failed ($p = 0.016$)
Equation S5-11: $\ln(\varepsilon') = 5.552 + (0.00355 \times T) - (0.443 \times \ln f) + (0.0000269 \times T^2) + (0.0372 \times \ln f^2) - (0.00140 \times T \times \ln f)$
$R^2 = 0.976$
Normality Test: Passed ($p = 0.051$)
Constant Variance Test: Passed ($p = 0.251$)
Equation S5-12: $1/\varepsilon' = -0.000903 - (0.0000504 \times T) + (0.00494 \times \ln f) - (0.000000317 \times T^2) - (0.000402 \times \ln f^2) + (0.0000201 \times T \times \ln f)$
$R^2 = 0.981$
Normality Test: Passed ($p = 0.137$)
Constant Variance Test: Failed ($p = 0.032$)
Equation S5-13: $\varepsilon'' = 2340.995 + (13.582 \times T) - (896.688 \times \ln f) + (0.0174 \times T^2) + (80.816 \times \ln f^2) - (2.210 \times T \times \ln f)$
$R^2 = 0.964$

Normality Test: Failed (p = 0.006)
Constant Variance Test: Failed (p = < 0.001)
Equation S5-14: $\sqrt{\varepsilon''}$ = 63.754 + (0.269 × T) - (19.051 × ln f) + (0.000113 × T^2) + (1.489 × ln f^2) - (0.0368 × T × ln f)
R^2 = 0.994
Normality Test: Passed (p = 0.214)
Constant Variance Test: Failed (p = 0.008)
Equation S5-15: ln(ε'') = 9.793 + (0.0169 × T) - (1.403 × ln f) - (0.00000389 × T^2) + (0.0553 × ln f^2) - (0.00113 × T × ln f)
R^2 = 0.999
Normality Test: Passed (p = 0.402)
Constant Variance Test: Passed (p = 0.391)
Equation S5-16: 1/ε''= 0.0525 + (0.000245 × T) - (0.0292 × ln f) + (0.000000199 × T^2) + (0.00438 × ln f^2) - (0.0000852 × T × ln f)
R^2 = 0.993
Normality Test: Passed (p = 0.048)
Constant Variance Test: Passed (p = 0.313)
